# Metabolomics in Thoracic Oncology from Biomarker Discovery to Therapeutic Targeting: A Narrative Review

**DOI:** 10.3390/cancers18213582

**Published:** 2026-11-07

**Authors:** Mohamed A. Mahmoud, Gaurav Sharma, Inderpal S. Sarkaria, Matthew R. Porembka, Ralph J. DeBerardinis, Kemp H. Kernstine

**Affiliations:** 1 Department of Cardiovascular and Thoracic Surgery, University of Texas Southwestern Medical Center, Dallas, TX 75390, USA; mohamedatefmohamed.mahmoud@utsouthwestern.edu (M.A.M.); gaurav.sharma@utsouthwestern.edu (G.S.); inderpal.sarkaria@utsouthwestern.edu (I.S.S.); 2 Department of Surgery, University of Texas Southwestern Medical Center, Dallas, TX 75390, USA; matthew.porembka@utsouthwestern.edu; 3 Eugene McDermott Center for Human Growth and Development, University of Texas Southwestern Medical Center, Dallas, TX 75390, USA; ralph.deberardinis@utsouthwestern.edu; 4 Children’s Medical Center Research Institute, University of Texas Southwestern Medical Center, Dallas, TX 75390, USA; 5 Howard Hughes Medical Institute, University of Texas Southwestern Medical Center, Dallas, TX 75390, USA

**Keywords:** thoracic oncology, cancer metabolomics, stable-isotope tracing, metabolic flux analysis, tumor microenvironment, metabolic biomarkers, precision oncology, metabolism-targeted therapy

## Abstract

Thoracic malignancies show context-dependent metabolic plasticity that influences growth, immune evasion, and treatment response. This review examines how metabolomics, stable-isotope tracing, and metabolic flux analysis complement molecular profiling in lung, esophageal, pleural, and thymic cancers. We distinguish metabolite abundance from isotope enrichment and quantitative flux, and review analytical platforms, computational workflows, histology-specific programs, immunometabolism, biomarkers, and therapy. Lung squamous cell carcinoma often depends on glycolysis, lung adenocarcinoma shows substrate and spatial heterogeneity, and molecular subsets of small cell lung cancer have preclinical vulnerabilities in arginine metabolism and guanosine nucleotide synthesis. Hypoxia and metabolite exchange among malignant, stromal, and immune cells may shape therapeutic resistance. Biofluid and tissue studies support early detection and monitoring, but most circulating signatures and findings in rare thoracic tumors remain exploratory. Translation is limited by confounding, incomplete metabolite identification, small cohorts, and limited external validation. Trials of unselected metabolic inhibitors have largely failed. Two biomarker-selected strategies have advanced: arginine deprivation in non-epithelioid pleural mesothelioma, a population enriched for argininosuccinate synthetase 1 (ASS1) deficiency, and PRMT5 inhibition in tumors with MTAP deletion. These results suggest that success may depend on selecting patients by a demonstrated metabolic dependency rather than by the plausibility of the target. Progress requires harmonized multicenter workflows, paired tissue and biofluid sampling, rigorous validation, and integration with spatial, single-cell, multi-omic, and clinical data.

## Simple Summary

Cancers of the chest, including lung, esophageal, pleural, and thymic tumors, remain among the most difficult cancers to treat. These tumors rewire the way they use nutrients such as sugars, amino acids, and fats to grow, evade the immune system, and survive treatment. Metabolomics is a laboratory approach that measures these small molecules in tumors, blood, and other body fluids, and related methods can track how nutrients are used inside a living tumor. This review explains what these tools can and cannot show, summarizes what they have revealed about each type of chest cancer, and asks whether they can help detect cancer earlier, predict who will benefit from treatment, and guide drugs aimed at tumor metabolism. It also explains why most findings are not yet ready for routine patient care and what would be needed to change that.

## 1. Introduction

Thoracic malignancies comprise a biologically and clinically heterogeneous group of neoplasms. For the purposes of this review, the term encompasses malignancies of the lung, pleura, thymus, and esophagus, reflecting their frequent evaluation and management within multidisciplinary thoracic oncology. Lung and esophageal cancers contribute substantially to the global cancer burden, whereas pleural mesothelioma (PM) and thymic epithelial tumors (TETs) are less common [1,2,3,4]. The 2021 World Health Organization classification omits the former prefix “malignant” from pleural mesothelioma [3]. Despite their distinct histologic, molecular, and clinical characteristics, studies across these diseases indicate that metabolic phenotypes are shaped by interactions among oncogenic signaling, cellular lineage, nutrient availability, and the immune and stromal components of the tumor microenvironment (TME) [5,6,7,8,9,10]. These observations have prompted interest in biological frameworks that complement conventional histopathologic classification by examining metabolic reprogramming and its relationship to tumor progression and therapeutic response, as a complement to histopathologic classification. Lung cancer was the leading cause of cancer-related mortality worldwide in 2022, accounting for approximately 1.8 million deaths [2]. Lung cancers are commonly grouped into non-small cell lung cancer (NSCLC), which accounts for approximately 85% of cases, and small cell lung cancer (SCLC), which accounts for approximately 15% [8,11]. The current World Health Organization classification additionally recognizes several less common pulmonary tumor categories [3]. Adenocarcinoma and squamous cell carcinoma are the predominant NSCLC histologic types, whereas large cell carcinoma is uncommon and represents a diagnosis of exclusion that should be restricted to adequately sampled resection specimens [3]. These histologic entities exhibit substantial differences in molecular architecture and metabolic behavior. Experimental and proteogenomic studies have identified a distinct glycolytic phenotype and associated metabolic vulnerabilities in subsets of lung squamous cell carcinoma [6,7]. SCLC, by contrast, is a highly aggressive neuroendocrine carcinoma characterized by rapid proliferation, early systemic dissemination, near-universal functional inactivation of TP53 and RB1, and extensive lineage plasticity [8]. MYC activation can promote progression toward a variant neuroendocrine-low state with distinct metabolic and therapeutic vulnerabilities [9].

Beyond the lung parenchyma, PM is an aggressive malignancy arising from the pleural mesothelium and is predominantly associated with asbestos exposure [3,4]. TETs are rare prevascular mediastinal neoplasms that include thymomas, thymic carcinomas, and thymic neuroendocrine tumors, which represent distinct pathologic and clinical entities [3]. Exploratory metabolomic studies of serum from mice bearing two PM patient-derived xenografts and of a small TET tissue cohort have identified alterations in glycolysis, tricarboxylic acid (TCA) cycle activity, amino acid and nucleotide metabolism, glutaminolysis, and redox homeostasis [10,12]. These preliminary studies nominate pathways for further investigation but do not establish clinically validated biomarkers or therapeutic targets.

Esophageal cancer also imposes a substantial global health burden and comprises two predominant histologic types: esophageal squamous cell carcinoma (ESCC) and esophageal adenocarcinoma (EAC) [1,13]. ESCC predominates globally and is especially common in high-incidence regions of Eastern Asia and eastern and southern Africa, whereas EAC incidence has increased markedly in Western populations and is associated with gastroesophageal reflux disease, Barrett esophagus, and obesity [13]. Metabolic and immunologic remodeling of the esophageal TME may contribute to disease progression and therapeutic resistance [5]. Recent single-cell analysis of ESCC has also identified immune-cell states associated with response to neoadjuvant immunochemotherapy, illustrating the potential influence of the TME on treatment response [14]. This biological diversity is itself a reason to evaluate metabolomics and metabolic flux analysis as complementary ways of identifying tumor-specific metabolic programs, candidate biomarkers, and treatment vulnerabilities.

Targeted therapies and immune checkpoint inhibitors have improved outcomes in selected thoracic cancer populations, particularly in biomarker-defined NSCLC [11] and with checkpoint inhibitors, in pleural mesothelioma [4]; however, prognosis remains poor for many patients with advanced disease [1,4,8]. Metabolic plasticity and an immunosuppressive TME may contribute to therapeutic resistance in specific histologic and molecular contexts, but they are not the sole determinants of disease progression or survival [5,8,9,15,16,17]. Modern tumor classification remains grounded in histopathology and increasingly incorporates genomic information [3,11], together with transcriptomic and proteomic data [7] to define oncogenic pathways and guide targeted treatment. Although these platforms have yielded essential biological and clinical insights, they do not directly measure biochemical pathway activity or metabolic flux. Transcript and protein abundance may not correspond directly to enzymatic activity because of post-transcriptional and post-translational regulation, substrate availability, nutrient and oxygen conditions, and interactions with stromal and immune cells [7,16]. Metabolomic measurements can therefore complement established molecular platforms by providing information about the downstream biochemical state produced by these regulatory processes [15,16].

Metabolomics has emerged as a systems-level approach for investigating thoracic tumor biology. Metabolomics involves the systematic measurement and analysis of small-molecule metabolites, generally with molecular masses below approximately 1500 Da, in cells, tissues, biofluids, and other biological specimens [15,16]. These molecules include nutrients, pathway intermediates, signaling mediators, hormones, xenobiotics and drugs, and metabolic end products spanning diverse physicochemical properties and concentrations. Because metabolite pool sizes reflect the combined influence of genomic, transcriptional, translational, enzymatic, environmental, and systemic regulation, metabolomics can provide complementary information about cellular and organismal biochemical states [15,16]. However, metabolite abundance is also influenced by diet, medications, comorbidities, microbiome activity, systemic organ metabolism, and specimen collection and processing. Consequently, a circulating metabolic profile should not automatically be interpreted as tumor-specific [15,18]. Steady-state metabolomics measures metabolite pool sizes and does not, by itself, quantify pathway turnover or metabolic flux. Quantitative flux estimation requires appropriately designed stable-isotope-tracing experiments, labeling data at isotopic steady state or resolved over time, and computational modeling [16,19].

In precision oncology, metabolomics is increasingly used as a research and translational platform but is not yet routinely incorporated into clinical decision-making [15,16,20]. Metabolic profiling is being investigated for candidate biomarker discovery, disease detection, phenotypic stratification, risk assessment, and longitudinal monitoring of treatment response [15,16]. Most reported signatures, however, require further analytical standardization, independent validation, prospective clinical evaluation, and demonstration of added value beyond established diagnostic and prognostic methods before routine implementation [15,18,20,21]. This framework is particularly relevant in oncology, where metabolic reprogramming can support biosynthesis, redox homeostasis, survival under stress, immune evasion, and adaptation to nutrient- and oxygen-limited microenvironments [15,16,17]. Accordingly, the principal current value of metabolomics lies in its integration with genomic, transcriptomic, proteomic, clinical, and metabolic-flux data rather than its use as a stand-alone precision-oncology platform.

### Review Scope, Literature Search, and Evidence Appraisal

This article is a narrative review rather than a systematic review or meta-analysis, and its preparation was guided by the SANRA (Scale for the Assessment of Narrative Review Articles) quality criteria [22]. We identified the literature through PubMed searches covering articles published from January 2000 to October 2026, with the final update performed during revision in October 2026. Four search strings combined a common block of thoracic-malignancy terms (lung cancer, esophageal cancer, pleural mesothelioma, and thymic epithelial tumors) with concept blocks for (S1) metabolomics, stable-isotope tracing, metabolic flux analysis, and metabolic imaging; (S2) the tumor microenvironment and immunometabolism; (S3) metabolic biomarkers in tissue and biofluids; and (S4) metabolism-targeted therapy. The complete strings are given in Appendix A (Table A1). Reference lists of included articles and of key reviews were screened to identify additional sources, and registered trials of metabolism-targeted agents were identified and their status checked on ClinicalTrials.gov (https://clinicaltrials.gov/, accessed 10 October 2026). Sources for tumor classification, epidemiology, and methodological standards (for example, the WHO classification [3], GLOBOCAN [2], and the ISO 23118, mQACC, and Metabolomics Standards Initiative standards cited in Section 6.5 and Section 7) were identified separately.

We prioritized peer-reviewed human studies, in vivo isotope-tracing studies, and randomized or prospective clinical trials, supplemented by mechanistic preclinical work where it established biological rationale. Studies were eligible if they reported metabolomic, stable-isotope-tracing, metabolic-imaging, or metabolism-targeted therapeutic data relevant to lung, esophageal, pleural, or thymic malignancies; conference abstracts without peer-reviewed data, non-English articles, and studies without extractable metabolic data were excluded. Studies from other tumor types or from non-tumor-bearing models were included where they provided the principal mechanistic, methodological, or clinical evidence for a concept discussed here (for example, systemic isotope-tracing studies in mice and the CANTATA trial in renal cell carcinoma), and they are identified as such in the text. Articles were selected by the authors on the basis of relevance and methodological quality. Because the available evidence differs substantially in maturity across these diseases, no formal risk-of-bias scoring or quantitative synthesis was undertaken, consistent with the narrative design.

To help the reader weigh the strength of the evidence, we distinguish throughout between (i) mechanistic and in vitro findings, (ii) animal-model data, (iii) human observational or cross-sectional biomarker associations, (iv) independently or prospectively validated biomarkers, and (v) randomized controlled-trial evidence. A metabolic feature is described as a dependency or vulnerability only when it is supported by genetic or pharmacological perturbation; altered enzyme expression or metabolite abundance alone is described as an association. Individual metabolic biomarkers and therapeutic strategies are further classified as clinically supported (benefit shown in a randomized trial), promising but preliminary (human data with independent validation or early-phase clinical activity), preclinical (animal or in vitro data only), hypothesis-generating (human or model associations without independent validation), or insufficiently supported (no efficacy data, or negative or terminated trials). This classification is applied explicitly in Section 5.5 and in the summary tables of Section 7 and Section 10, and is intended to prevent exploratory findings, particularly in the rare thoracic tumors, where cohorts are small, from being read as established clinical applications.

The figures are schematic. Basic outlines were prepared in BioRender, and the illustrations were then produced with ChatGPT (GPT-6 Astra, medium; OpenAI, 2026) from those outlines and the authors’ written descriptions. The authors checked every depicted element against the references cited in each legend and corrected or removed elements that the cited evidence did not support.

## 2. Fundamentals of Metabolic Analysis: Metabolomics vs. Isotope Tracing

The distinction between steady-state metabolomics, isotope-enrichment analysis, and metabolic flux analysis (MFA) is central to interpreting tumor metabolism (Table 1). Steady-state metabolomics measures relative or absolute metabolite abundances and identifies pathway-associated changes, whereas stable-isotope tracing reveals the incorporation and fate of labeled nutrients. Neither metabolite abundance nor isotope enrichment alone, however, establishes pathway turnover; quantitative flux estimation generally requires appropriate tracer design, time-resolved or isotopic steady-state labeling data, independent rate constraints, and computational modeling [19]. This distinction is particularly important when interpreting the Warburg effect. Although aerobic glycolysis remains a foundational feature of cancer metabolism, contemporary profiling has revealed alterations in oxidative phosphorylation (OXPHOS), TCA-cycle metabolism, glutaminolysis, serine biosynthesis, lipid metabolism, autophagy, and nutrient exchange within the TME [5,17]. Increased glycolytic flux should not, however, be equated with uniformly elevated adenosine triphosphate (ATP) production or energy demand.

Using in vivo isotope-tracer infusions, mass spectrometry, and kinetic modeling, Bartman and colleagues quantified glycolytic and TCA-cycle fluxes and estimated ATP-production rates in healthy mouse tissues and multiple mouse tumor models [23]. Across five primary solid-tumor models in mice, TCA-cycle flux and model-derived total ATP-production rates were lower than in the corresponding healthy mouse tissues, and increased glycolytic flux was insufficient to compensate energetically for reduced oxidative metabolism [23]. The same study reported that TCA-cycle flux was increased in lung metastases of breast cancer relative to matched primary orthotopic tumors, suggesting that suppressed oxidative flux reflects the primary-tumor environment rather than being an intrinsic feature of malignancy [23]. Anatomical site and cancer stage therefore shape metabolic phenotype alongside lineage and genotype. That distinction matters in thoracic oncology, where the lung is both a primary site and a common site of metastases, and where metabolism-targeted agents have been tested mainly in metastatic disease. In these mouse models, the findings do not revise the classical interpretation of the Warburg effect; rather, they challenge the assumption that proliferating tumors must be hypermetabolic in terms of total ATP production and demand. In mouse pancreatic tumors, reduced protein synthesis, a major energetic cost of the differentiated exocrine pancreas, accompanied the lower ATP-production rate [23]. This observation supports the authors’ proposal that loss of energetically demanding tissue-specific functions can reduce ATP requirements, thereby permitting tumor growth despite comparatively limited ATP production. At present, it is unknown how fully these findings translate to human disease. In vivo isotope-tracing studies in patients with NSCLC have demonstrated nutrient utilization, substantial metabolic heterogeneity, and an association between TCA cycle metabolism and poor clinical outcomes [24,25,26]. Measurements of absolute glycolytic and TCA-cycle fluxes coupled to total ATP-production rates remain, to our knowledge, unavailable for human thoracic tumors [27].

**Figure 1:**
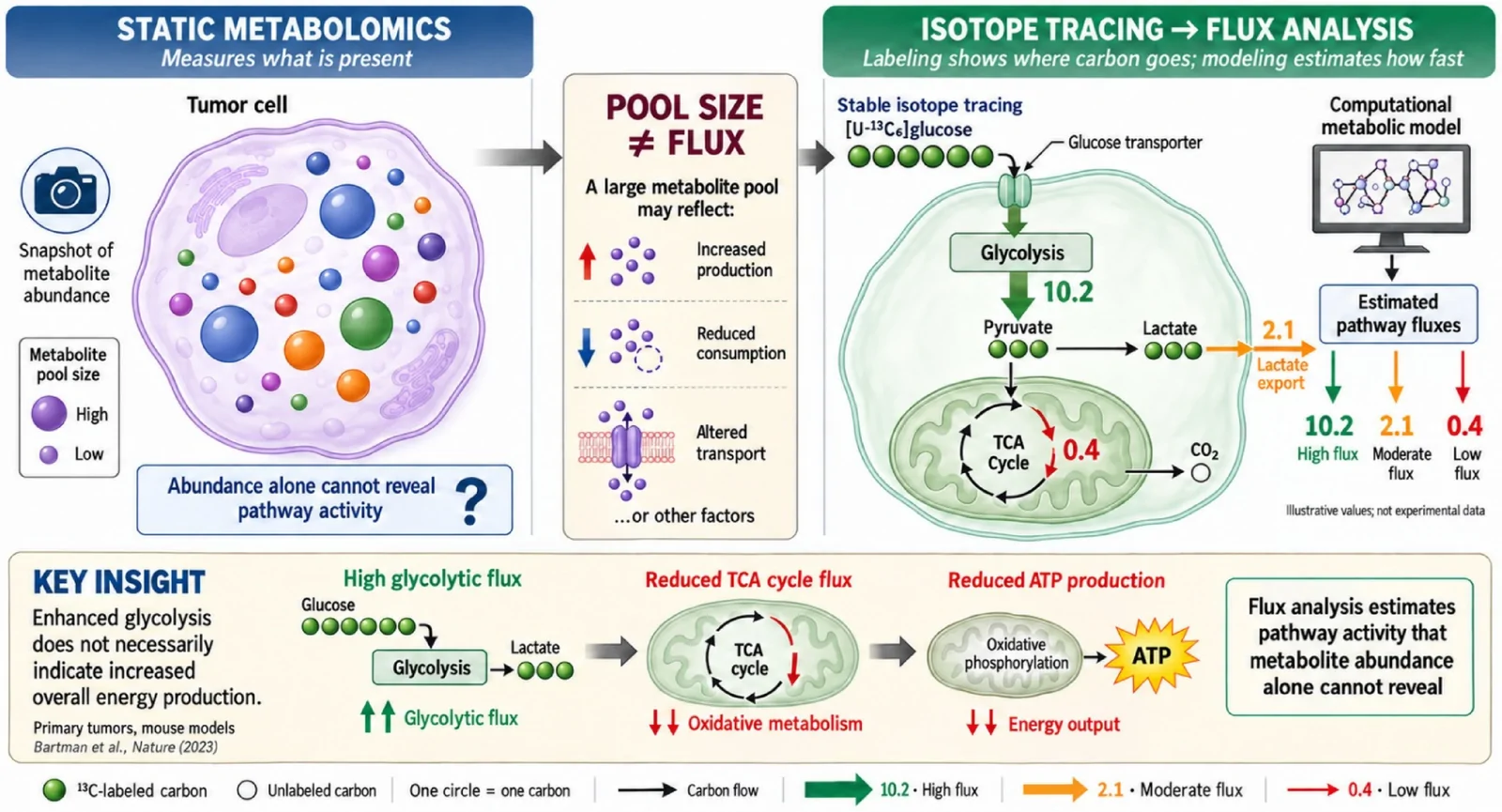
Static metabolomics versus isotope tracing and flux analysis. Static metabolomics (**left**) measures metabolite pool sizes, but cannot, on its own, reveal pathway activity, because a large pool may reflect increased production, reduced consumption, altered transport, or other factors. Fluxomics, stable isotope-resolved metabolomics (SIRM) and ^13^C metabolic flux analysis (^13^C-MFA), instead measure how carbon flows through the network (**right**). Infused ^13^C-glucose is traced through glycolysis and the TCA cycle, and the labeling data are fitted to a computational metabolic model to yield quantitative pathway fluxes (illustrative values: high 10.2, moderate 2.1, low 0.4). The key insight (**bottom**) is that enhanced glycolysis does not necessarily indicate increased overall energy production: primary tumors can show high glycolytic flux alongside reduced TCA-cycle flux and reduced ATP output, so flux analysis uncovers true metabolic activity that metabolite abundance alone conceals. Filled circles denote ^13^C-labeled carbon and open circles denote unlabeled carbon; arrow color indicates flux magnitude (green, high; orange, moderate; red, low). Upward and downward arrows indicate increases and decreases; circle size in the left panel denotes relative metabolite pool size, as shown in the figure key; and the question mark denotes pathway activity that abundance alone cannot resolve. Based on [19,23,27]. (Schematic; not to scale. Numerical flux values are illustrative).

The metabolome is dynamic. Metabolite concentrations are transient snapshots set by the balance of synthesis, degradation, utilization, transport, and environmental exchange [15,16]. Consequently, pathway activity cannot be reliably inferred from metabolite pool sizes alone, because metabolite accumulation may reflect increased production, reduced consumption, altered transport, or impaired downstream metabolism. To overcome this limitation, stable isotope-resolved metabolomics (SIRM) and ^13^C metabolic flux analysis (^13^C-MFA) incorporate isotopic tracers into metabolic investigations, enabling direct assessment of carbon flow through biochemical networks (Figure 1). By supplying labeled substrates such as [U-^13^C]glucose, ^13^C-glutamine, pyruvate, lactate, alanine, palmitate, or acetate, investigators can follow isotope incorporation into downstream metabolites and, when labeling data are combined with an appropriate network model, estimate pathway utilization within complex metabolic systems [16,19]. For example, [1,2-^13^C_2_]glucose can distinguish glycolytic from pentose phosphate pathway activity, whereas [U-^13^C_5_]glutamine is widely used to assess TCA cycle metabolism, anaplerosis, and oxidative versus reductive glutamine utilization [19]. Fluxomic approaches integrate isotope-labeling data with network models of known reactions and carbon atom transitions. They thereby measure pathway activity and substrate use in vivo, which static abundance information cannot [16,19].

These methods demand rigorous standardization. Some enzymatic reactions occur within seconds, so metabolite concentrations can change within seconds of sampling. The critical step is therefore to minimize warm ischemia by quenching metabolism rapidly, usually by flash-freezing, so that measured concentrations reflect the living tissue. No single analytical platform captures the entire metabolome (Figure 1). The analytes are chemically diverse and span millimolar to picomolar concentrations. Comprehensive coverage, therefore, requires a combination of high-resolution spectroscopic platforms, primarily nuclear magnetic resonance (NMR) spectroscopy and mass spectrometry (MS). Depending on the experimental objective, acquisition strategies follow either unbiased, untargeted profiling for global screening and novel hypothesis generation, or targeted quantification using internal standards for the precise measurement of predefined biochemical pathways. Public reference registries such as the Human Metabolome Database help interpret these multi-platform datasets, because they integrate curated chemical, clinical, and biochemical data [16].

## 3. Metabolic Reprogramming in Thoracic Malignancies

Metabolic reprogramming in thoracic malignancies arises through multiple convergent mechanisms, including genetic alterations that directly affect metabolic regulation, such as loss of KEAP1 and consequent NRF2 activation in subsets of NSCLC [28], as well as secondary metabolic consequences of oncogenic signaling and dynamic selective pressures imposed by hypoxia, nutrient limitation, and therapeutic stress [15,17]. These alterations emerge through both cancer cell-intrinsic mechanisms, including oncogene activation and tumor-suppressor loss, and cancer cell-extrinsic influences originating from the TME. Among the latter, hypoxia is a major driver of metabolic adaptation, mediated in part by hypoxia-inducible factor-1α (HIF-1α), which coordinates transcriptional programs that support survival and proliferation under oxygen-limited conditions and contribute to intratumoral metabolic heterogeneity [17]. The resulting metabolic phenotype reflects the context-dependent plasticity of malignant cells, which rewire bioenergetic, biosynthetic, and redox pathways to sustain growth, survival, and adaptation. This reprogramming is further shaped by microenvironmental factors, including stromal composition, immune-cell infiltration, nutrient availability, oxidative stress, and tissue architecture [15,16,17].

At the molecular level, receptor tyrosine kinase signaling activates the PI3K–AKT pathway, promoting glucose uptake and glycolytic metabolism through regulation of glucose transporters and key glycolytic enzymes while simultaneously enhancing mTORC1 signaling. Under hypoxic conditions, stabilization of HIF-1α induces the expression of glucose transporter 1 (GLUT1), glucose transporter 3 (GLUT3), hexokinase 2 (HK2), and 6-phosphofructo-2-kinase/fructose-2,6-bisphosphatase 3 (PFKFB3), while increasing pyruvate dehydrogenase kinase activity, thereby limiting pyruvate oxidation and favoring lactate production. Oncogenic RAS signaling further promotes glycolysis and pentose phosphate pathway activity to support anabolic metabolism, nucleotide biosynthesis, and redox homeostasis [15]. These metabolic programs are not fixed consequences of genetic alterations. They are continuously reshaped by nutrient availability, hypoxia, stromal interactions, immune-mediated pressures, oxidative stress, and therapeutic interventions, resulting in substantial metabolic heterogeneity both within and between tumors [5,17].

These general mechanisms manifest differently across thoracic tumor lineages. Lung squamous cell carcinoma (LUSC) frequently exhibits high GLUT1 expression and glycolytic flux, resulting in greater dependence on glucose metabolism than lung adenocarcinoma (LUAD) and selective sensitivity to glycolytic inhibition in preclinical models [6]. By contrast, isotope-tracing studies in human NSCLC have demonstrated substantial inter- and intratumoral variation in glucose oxidation and shown that circulating lactate can contribute carbon to the TCA cycle [24,25]. Systemic tracer studies in mice established the physiological context for these observations, demonstrating that circulating lactate turnover exceeds that of glucose and that glycolysis and the TCA cycle are partially uncoupled at the level of lactate [29]. Driver genotype introduces an additional layer of heterogeneity; for example, proteogenomic analysis of LUAD identified enrichment of amino acid-metabolism proteins in STK11-mutant tumors [30]. In SCLC, MYC-driven tumors exhibit a distinct metabolic phenotype characterized by dependence on arginine-regulated pathways, including polyamine biosynthesis and mTOR signaling, whereas MYCL-driven tumors display different metabolic requirements [31]. Exploratory studies in TETs and PM models have also identified alterations in glycolysis, glutamine and redox metabolism, and circulating metabolite profiles; however, these preliminary findings should be regarded as hypothesis-generating rather than evidence of validated biomarkers or therapeutic targets [10,12]. Beyond carbohydrate and glutamine metabolism, methionine and one-carbon metabolism constitute a further axis of genotype-associated dependency. Methionine-cycle activity driven by methionine adenosyltransferase 2A (MAT2A) has been identified as a metabolic dependency of lung tumor-initiating cells [32], LKB1 loss links serine and one-carbon metabolism to S-adenosylmethionine availability and DNA methylation in pancreatic models [33], and competition for methionine between tumor cells and CD8+ T cells has been shown to constrain T-cell effector function in non-thoracic tumor models [34]. Homozygous deletion of methylthioadenosine phosphorylase (MTAP), which arises through co-deletion with CDKN2A at 9p21.3 in a substantial minority of NSCLC, causes accumulation of methylthioadenosine and consequent partial inhibition of protein arginine methyltransferase 5 (PRMT5), creating a synthetic-lethal dependency that has since been translated into clinical agents (Section 10) [35,36,37]. These examples show that metabolic reprogramming in thoracic malignancies depends on histologic lineage, driver genotype, local nutrient availability, and tissue context.

## 4. Tumor Microenvironment and Immunometabolism

These metabolic adaptations become particularly consequential within the TME, which functions not as a passive structural scaffold but as a metabolically interconnected ecosystem composed of malignant cells, cancer-associated fibroblasts (CAFs), cancer-associated adipocytes (CAAs), immune populations, vascular networks, extracellular matrix, and soluble mediators that collectively regulate tumor progression, metastasis, immune escape, and treatment response [17]. Within this environment, malignant, stromal, and immune cells draw on a shared and limited supply of glucose, glutamine, oxygen, amino acids, and lipids, while malignant cells reshape stromal and immune metabolism to establish an immunosuppressive niche [17]. Nutrient uptake is nonetheless cell-type-specific: in murine tumors, myeloid cells took up the most glucose and cancer cells the most glutamine, so the TME is better described as programmed nutrient partitioning than as simple competition [38]. Consequently, metabolites such as lactate, amino acids, adenosine, and free fatty acids function not merely as nutrient substrates but also as signaling mediators capable of regulating stromal behavior, angiogenesis, epithelial–mesenchymal transition, and immune-cell fate [5]. Much of the mechanistic evidence summarized in this section derives from murine models and non-thoracic tumors; where direct measurements in human thoracic tumors exist, they are identified as such.

Among stromal populations, CAFs are major regulators of metabolic adaptation and immune remodeling within the TME. Under the influence of transforming growth factor-β (TGF-β), oxidative stress, and hypoxia, CAFs frequently undergo metabolic reprogramming characterized by enhanced glycolysis and increased expression of glycolytic enzymes, including HK2, 6-phosphofructo-2-kinase/fructose-2,6-bisphosphatase 3 (PFKFB3), pyruvate kinase M2 (PKM2), and lactate dehydrogenase A (LDHA). This phenotype has been proposed to underlie the reverse Warburg effect, in which CAFs export lactate through monocarboxylate transporter 4 (MCT4) while neighboring cancer cells import lactate via monocarboxylate transporter 1 (MCT1) to support oxidative metabolism and TCA cycle activity [39]. This model rests principally on co-culture systems and on compartment-specific transporter expression in tissue sections. Because transporter abundance is not a flux measurement, as Section 2 explains, the direction and magnitude of net stromal-to-tumor lactate transfer in human tumors remain to be established by in vivo isotope tracing. Beyond serving as a metabolic substrate, CAF-derived lactate contributes to extracellular acidification and promotes an immunosuppressive microenvironment by impairing cytotoxic CD8+ T-cell and natural killer (NK)-cell function while supporting regulatory T-cell (Treg) persistence [17].

CAAs represent an additional metabolic component of the TME, particularly in tumors located adjacent to adipose-rich tissues. Tumor-derived signals induce extensive lipolytic remodeling characterized by activation of adipose triglyceride lipase, hormone-sensitive lipase, and monoacylglycerol lipase, resulting in sustained release of free fatty acids and glycerol. These metabolites are subsequently acquired by tumor cells through transporters such as CD36 and utilized to support mitochondrial β-oxidation, survival under nutrient stress, and metastatic progression [17]. Simultaneously, lipid-rich microenvironments impair dendritic-cell antigen presentation, promote suppressive myeloid-derived suppressor cell (MDSC) phenotypes, stabilize PPARγ-dependent Tregs, and contribute to metabolic dysfunction of effector lymphocytes. Evidence for this axis derives mainly from experimental models of tumors growing within adipose-rich tissue, and it has not been examined directly in thoracic tumors.

Among the metabolites shaping these interactions, lactate has emerged as a central regulator of tumor progression and immune suppression. Once regarded primarily as a byproduct of glycolysis, lactate is now recognized as an important metabolic fuel, signaling molecule, and regulator of immune-cell function within solid tumors [5,17]. Elevated lactate concentrations acidify the TME and impair antitumor immunity through multiple mechanisms, including inhibition of T-cell proliferation and effector function, disruption of metabolic fitness, and promotion of immunosuppressive cell populations. Lactate additionally promotes M2-like macrophage polarization through GPR132 signaling in murine breast cancer models [40]. In human T cells in vitro, it blocks lactate export through MCT1, which disturbs T-cell metabolism and reduces cytokine production and cytotoxicity [17,41]. These effects favor the expansion and maintenance of Tregs and MDSCs while suppressing NK-cell function and cytokine production: in murine models, tumor-derived lactate increased MDSC numbers and impaired NK-cell cytotoxicity [42].

Lactate is now recognized to act not only as a fuel and a receptor ligand but also as a substrate for post-translational modification. Histone lysine lactylation couples glycolytic activity to chromatin state and drives a distinct late-phase transcriptional program in macrophages [43]. In NSCLC, H3K18 lactylation has been reported to promote immune escape, suggesting a mechanism by which sustained glycolysis could produce durable transcriptional reprogramming that extracellular acidification alone would not explain [44]. Whether lactylation contributes materially to immunotherapy resistance in thoracic tumors in vivo, and whether it is pharmacologically tractable, remain open questions.

Hypoxia further amplifies these suppressive programs through HIF-1α-dependent metabolic reprogramming. Reduced oxygen availability shifts cellular metabolism toward glycolysis, enhancing lactate production and extracellular acidification while simultaneously promoting vascular endothelial growth factor A (VEGFA) signaling [17]. It also promotes adenosinergic immunosuppression through CD39- and CD73-dependent adenosine generation and A2A and A2B receptor signaling [17,45,46]. Hypoxia, additionally, promotes T-cell exhaustion, supports M2-like macrophage polarization, and facilitates the accumulation of immunosuppressive Treg populations, thereby linking metabolic stress directly to immune dysfunction and therapeutic resistance.

These principles are particularly evident in thoracic malignancies such as ESCC, where single-cell RNA sequencing integrated with T-cell receptor sequencing has identified stromal and immune cell populations associated with response to neoadjuvant immunochemotherapy [14]. Before treatment, CXCL13+ exhausted CD8+ T cells were more abundant in responders and were associated with better response; after treatment, these cells showed a progenitor-like exhaustion profile in responders but a terminally exhausted profile in non-responders, whose tumors were also enriched for activated TNFRSF4+CD4+ Tregs, LRRC15+ fibroblasts, and SPP1+ macrophages associated with an immunosuppressive microenvironment. Within these niches, ligand–receptor analysis predicted that fibroblast populations, including MMP11+ tissue CAFs and LRRC15+ CAFs, signal through LGALS9 to CD44-expressing TNFRSF4+CD4+ Tregs; this interaction was inferred computationally rather than demonstrated. These data are transcriptomic; the metabolic state of these cell populations was not measured directly. Direct metabolic evidence from human thoracic tumors is more limited but emerging. In resected, treatment-naive ESCC, metabolomic (136 tumor–normal pairs) and proteomic (127 tumors and 128 normal tissues) profiling identified an immunosuppressive subtype marked by creatine accumulation and hexokinase 3 (HK3) deficiency, and creatine and HK3 loss promoted M2-like macrophage polarization in mouse models [47]. In patients with NSCLC, intraoperative [U-^13^C]lactate infusion showed that human tumors use circulating lactate as a TCA-cycle fuel [24], confirming lactate exchange between tumor and circulation in patients, although its immune consequences have not been measured in human thoracic tumors.

**Figure 2:**
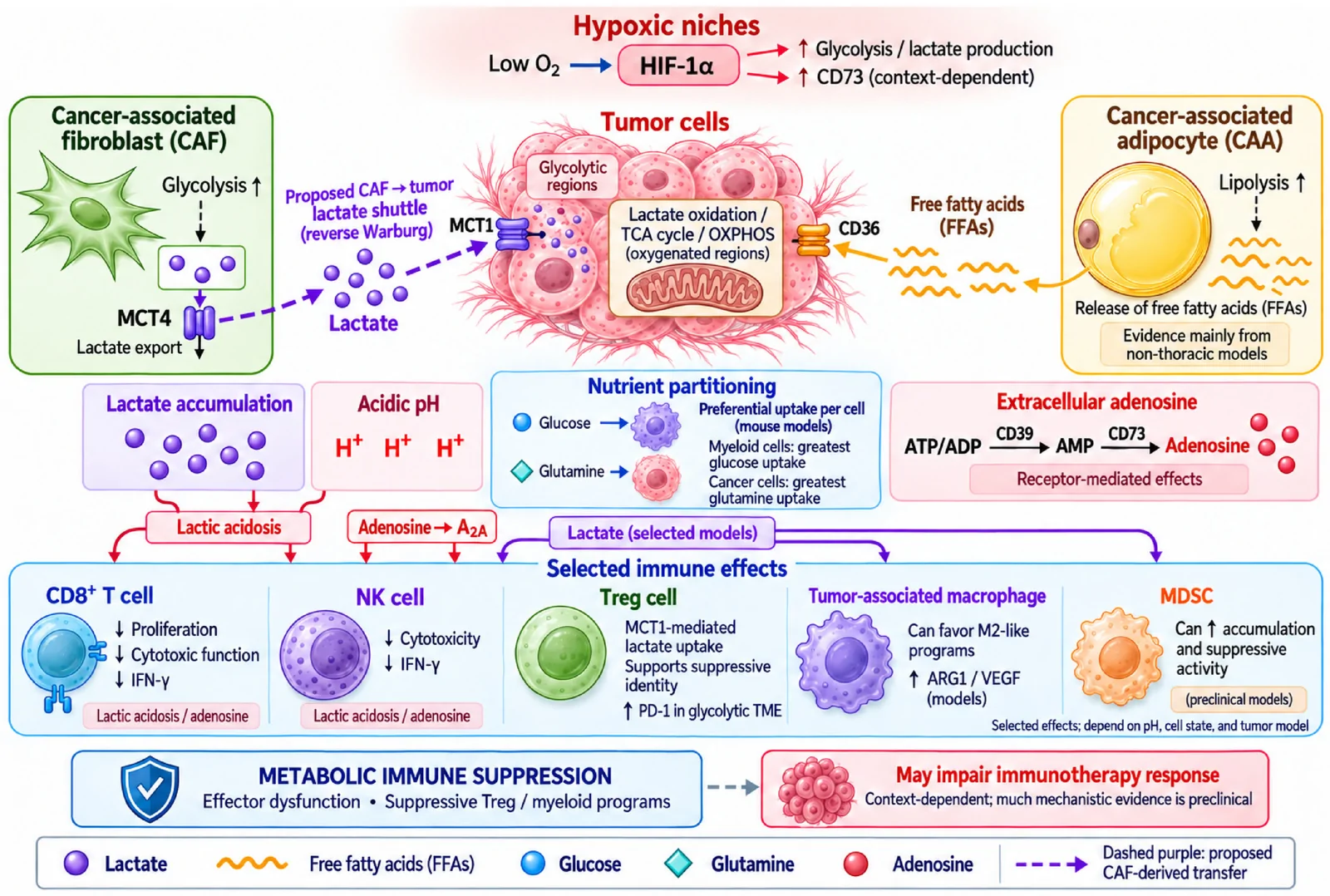
Metabolic crosstalk in the tumor microenvironment and its proposed effects on immune function. Hypoxia stabilizes HIF-1α, promoting glycolysis and lactate production and, in a context-dependent manner, inducing the adenosine-generating ectoenzyme CD73 [46]. Cancer-associated fibroblasts (CAFs, (**left**)) upregulate glycolysis and export lactate via MCT4; tumor cells may import it via MCT1 to fuel oxidative metabolism (TCA cycle/OXPHOS). This proposed “reverse Warburg” arrangement [39] has not, to our knowledge, been confirmed by in vivo isotope tracing in human tumors. Cancer-associated adipocytes (CAAs, (**right**)) undergo lipolysis, releasing free fatty acids taken up through CD36 [17] to support tumor-cell fatty-acid oxidation [56]. In murine tumors, nutrient uptake is cell-type-specific, with myeloid cells taking up the most glucose and cancer cells the most glutamine [38]. Elevated lactate, extracellular acidification (H+), and adenosine accumulation act on immune cells (bottom): CD8+ T cells show reduced proliferation, cytotoxic function, and interferon-γ output [41,49]; NK cells lose cytotoxicity and cytokine production [42,49]; regulatory T cells (Tregs) take up lactate through MCT1, which increases their PD-1 expression [48]; macrophages skew to an immunosuppressive M2-like phenotype [40,57]; and myeloid-derived suppressor cells (MDSCs) accumulate [42]. These effects may impair the response to immunotherapy and other anticancer therapies. The key (bottom) identifies lactate, free fatty acids, glucose, glutamine, and adenosine, and the dashed purple arrow denotes the proposed CAF-derived lactate transfer. Solid arrows indicate the direction of metabolite flow or effect, and upward and downward arrows indicate increases and decreases. Most links shown derive from murine models and non-thoracic tumors (see Section 4) [17,40,48]. (Schematic; not to scale).

These stromal–immune interactions operate in parallel with broader metabolic alterations within the TME. Increased glucose uptake, lactate accumulation, and altered mitochondrial metabolism reshape immune-cell function and influence therapeutic response. Tregs import extracellular lactate through MCT1 (SLC16A1), which increases programmed cell death protein 1 (PD-1) expression through NFAT1-dependent signaling [17,48]. In highly glycolytic tumors, the resulting PD-1-high Tregs may be activated by PD-1 blockade and so limit its efficacy [48]. In murine melanoma, LDHA-associated lactic acid production suppressed T- and NK-cell surveillance, and tumors with low LDHA expression grew more slowly in immunocompetent hosts [49]. Similar mechanisms have been observed in LKB1-deficient LUAD, where MCT4-dependent lactate secretion contributes to immune evasion [50]. Resistance in thoracic malignancies may therefore arise not only from tumor-cell-intrinsic mechanisms but from metabolic and immunological crosstalk among the malignant, stromal, and immune compartments that remodel the TME (Figure 2) [5,14,17].

Genotype-defined metabolic states provide the clearest clinical link between metabolism and immunotherapy response in thoracic oncology. In KRAS-mutant lung adenocarcinoma, STK11/LKB1 loss was associated with substantially reduced benefit from PD-1 blockade, with objective response rates of 7.4% in STK11/LKB1-mutant tumors compared with 35.7% in TP53-co-mutant and 28.6% in KRAS-only tumors in the Stand Up To Cancer cohort of 174 patients, and with significantly shorter progression-free and overall survival than STK11/LKB1-wild-type tumors [51]. Proposed mechanisms, derived largely from preclinical models, include silencing of STING with attenuated innate immune sensing [52] and MCT4-dependent lactate secretion [50]. KEAP1 mutation, which activates the NRF2 antioxidant program and generates glutamine dependence in preclinical models [28], frequently co-occurs with STK11 loss; in two independent cohorts, STK11 and KEAP1 mutations were associated with worse outcomes of PD-(L)1 blockade in KRAS-mutant, but not KRAS-wild-type lung adenocarcinoma [53]. These associations derive from retrospective genomic cohorts rather than prospective biomarker-stratified trials, and whether the effect is of similar magnitude with contemporary first-line chemo-immunotherapy combinations has not been established prospectively. Nevertheless, they indicate that metabolic genotype is associated with immune response and provide the interpretive context for the trial results discussed in Section 10.

A further source of metabolic influence on the thoracic immune microenvironment originates outside the tumor altogether. Gut microbial composition modulates the efficacy of PD-1 blockade across epithelial tumors, and antibiotic exposure around the time of treatment initiation has been associated with inferior outcomes [54]. In advanced NSCLC specifically, intestinal Akkermansia muciniphila has been reported to predict clinical response to PD-1 blockade [55]. The microbiome is doubly relevant here: it is the largest modifiable source of host-derived circulating metabolites, and it is correspondingly a major uncontrolled confounder for the circulating metabolite signatures discussed in Section 7, few of which have been analyzed with respect to concurrent antibiotic exposure or dietary intake.

## 5. Distinct Metabolic Phenotypes Across Thoracic Tumor Histologies

**Figure 3:**
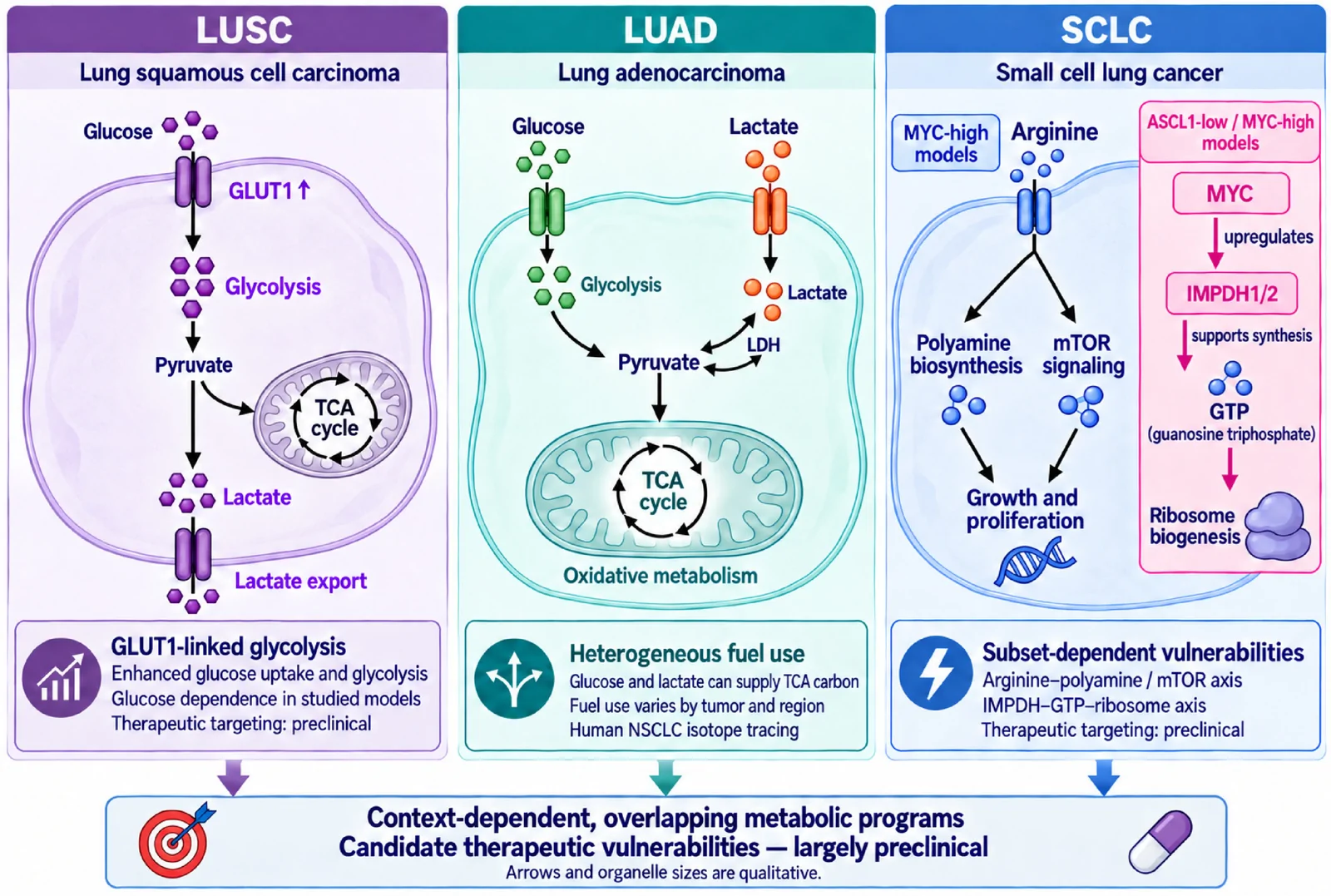
Metabolic programs across the major lung cancer subtypes. Schematic comparison of the dominant metabolic programs in lung squamous cell carcinoma (LUSC), lung adenocarcinoma (LUAD), and small cell lung cancer (SCLC). LUSC (**left**) shows GLUT1-linked glycolysis: high GLUT1-mediated glucose uptake feeds glycolysis and pyruvate, with lactate production and export, while pyruvate also enters the mitochondrial TCA cycle; glucose dependence has been shown in cell lines and xenografts [6]. LUAD (**center**) shows heterogeneous fuel use: both glucose and circulating lactate, the latter converted to pyruvate by lactate dehydrogenase (LDH), can supply carbon to the TCA cycle, and the balance between them varies between tumors and between regions of the same tumor. SCLC (**right**) shows subset-dependent vulnerabilities: in MYC-high models, arginine supports polyamine biosynthesis and mTOR signaling, whereas in an overlapping ASCL1-low/MYC-high subset, MYC upregulates IMPDH1/2, and these tumors depend on guanosine nucleotide synthesis, which supplies guanosine triphosphate (GTP) for ribosome biogenesis. These programs are context-dependent and partly overlapping, and the vulnerabilities they nominate remain largely preclinical: the LUSC and SCLC dependencies derive from cell-line and animal models [6,31,58,59], whereas the LUAD panel draws on intraoperative isotope tracing in patients with NSCLC [24,25]. Arrows indicate the direction of metabolite flow or regulation, and the upward arrow beside GLUT1 indicates increased expression. Arrows and organelle sizes are qualitative (schematic; not to scale).

This metabolic heterogeneity extends further across thoracic tumor histologies (Figure 3). Deep molecular and proteogenomic profiling indicates that pulmonary malignancies show divergent, histology-specific metabolic dependencies tailored to their respective cellular lineages and oncogenic configurations [6,7].

### 5.1. Lung Squamous Cell Carcinoma (LUSC)

LUSC exhibits one of the most consistently glycolytic metabolic phenotypes among thoracic malignancies, characterized by elevated glucose uptake, increased lactate production, enhanced extracellular acidification, and strong fluorodeoxyglucose (FDG) avidity [6,7]. A defining feature of LUSC is the marked overexpression of the facilitative glucose transporter GLUT1 (SLC2A1) at both the transcriptomic and proteomic levels, which supports increased glycolytic flux and contributes to the accumulation of extracellular lactate [6]. Functional studies in cell lines and xenografts have shown that this metabolic program creates a heightened dependence on glucose metabolism, rendering LUSC relatively sensitive to pharmacologic inhibition of glucose transport and glycolytic enzymes compared with lung adenocarcinoma [6].

Proteogenomic analyses further indicate that many of the metabolic features of LUSC are regulated through post-translational mechanisms in addition to transcriptional reprogramming [7]. Compared with matched normal lung tissue, the key glycolytic enzymes phosphoglycerate kinase 1 (PGK1) and pyruvate kinase M (PKM) show reduced acetylation at known inhibitory sites clustered around the enzymatic pocket, which implies increased activity, and this interpretation is supported by increased phosphorylation at known activating sites on the glycolytic regulators PFKFB3 (pS461) and PKM (pS37) [7]. Lactate handling in lung tumors is consistent with this phenotype, although squamous tumors were not analyzed separately. In human NSCLC specimens taken after intraoperative [U-^13^C]glucose infusion, MCT1, MCT4, LDHA, and LDHB were widely expressed, and all four tended to be higher in fragments with a greater lactate-to-3-phosphoglycerate labeling ratio [24].

Despite its strongly glycolytic phenotype, LUSC retains functional mitochondrial metabolism, although the supporting evidence derives from proteogenomic profiling and preclinical functional studies rather than from in vivo flux measurement in patients. Rather than representing a complete shift away from oxidative metabolism, glycolysis and mitochondrial pathways coexist and interact to support bioenergetic demands, anabolic growth, and redox homeostasis. Proteogenomic integration has revealed coordinated relationships between genomic alterations, post-translational enzyme regulation, and metabolic pathway activity. One histology-specific example is the association between SLC2A1 overexpression and enhanced glycolytic reliance in LUSC [6,7].

### 5.2. Lung Adenocarcinoma (LUAD)

In contrast to LUSC, LUAD exhibits substantial metabolic heterogeneity and greater metabolic flexibility. While many LUADs demonstrate increased glucose utilization, isotope-tracing studies have revealed considerable variability in the incorporation of glucose-derived carbon into TCA cycle intermediates, with some tumors relying extensively on alternative substrates, including circulating lactate, to support oxidative metabolism [24,25]. LUAD also depends less than LUSC on GLUT1-mediated glucose uptake [6], and a subset of LUAD tumors shows upregulated peroxisomal and PPAR signaling at the protein level [30].

Proteogenomic analysis links these metabolic differences to immune phenotype and to driver genotype rather than defining a metabolic taxonomy of its own. Unsupervised multi-omics clustering of LUAD resolved four subgroups defined by driver mutations, country, and sex; metabolic differences emerged within other comparisons [30]. Glycolysis, peroxisomal activity, and PPAR signaling were significantly upregulated at the protein level in immune-cold tumors, a pattern of interest because glycolysis has been implicated in immune evasion in other solid tumors but only marginally in LUAD [30]. Separately, STK11-mutant tumors were enriched for amino-acid-metabolism proteins associated with nitric oxide metabolic processes, which the authors interpret as suggesting perturbation of the urea cycle in this genotype [30]. These are protein- and transcript-level associations; the study included no metabolomic measurements, so the corresponding metabolite concentrations and pathway fluxes remain unmeasured. Moreover, metabolic phenotypes differ by histologic subtype. Acinar and non-mucinous adenocarcinomas tended to show low lactate-to-3-phosphoglycerate labeling ratios, whereas papillary and solid adenocarcinomas, and large-cell neuroendocrine carcinomas in the same NSCLC series, showed higher ratios, consistent with greater uptake of circulating lactate. Tumors with high ratios also tended to show high FDG uptake, and KRAS-mutant tumors were more heterogeneous [24]. High glucose contribution to the TCA cycle has in turn been associated with metastasis and early death [26].

### 5.3. Small Cell Lung Cancer (SCLC)

SCLC is a highly aggressive neuroendocrine malignancy characterized by rapid proliferation, early dissemination, and marked lineage plasticity [8,9]. Although near-universal functional inactivation of TP53 and RB1 provides a common genomic background, variation in lineage transcription factors and MYC-family activity gives rise to biologically and metabolically distinct tumor states [8,9,31,58]. SCLC is commonly classified into four transcriptional subtypes. SCLC-A, SCLC-N and SCLC-P are defined by relative expression of ASCL1, NEUROD1 and POU2F3 respectively [60,61]. The fourth subtype was originally defined by YAP1 expression [60] and was subsequently redefined as an inflamed subtype, SCLC-I, with low expression of all three lineage programs and an immune-related gene signature [61]. The metabolic phenotypes described below were defined principally by MYC-family activity rather than within this framework, and the correspondence between the metabolic and transcription-factor taxonomies has not been systematically established; mapping arginine- and guanosine-nucleotide-associated phenotypes onto SCLC-A/N/P/I remains an open question. Comparative metabolomic profiling of MYC-driven and MYCL-driven SCLC models identified distinct metabolic programs, with MYC-driven tumors exhibiting preferential dependence on arginine-regulated pathways, including polyamine biosynthesis and mTOR signaling. Arginine depletion with pegylated arginine deiminase suppressed MYC-driven tumor growth in genetically engineered mouse models, human cell-line xenografts, and one MYC-high patient-derived xenograft (PDX), identifying a preclinical subtype-associated vulnerability [31].

An overlapping ASCL1-low/MYC-high SCLC subset was characterized by elevated guanosine nucleotide abundance and increased expression of the guanosine-biosynthetic inosine monophosphate dehydrogenase (IMPDH) isoforms IMPDH1 and IMPDH2, resulting in dependence on guanosine nucleotide synthesis [58]. IMPDH inhibition suppressed ASCL1-low/MYC-high SCLC growth in cultured cells and xenografts and improved survival when combined with chemotherapy in genetically engineered mouse models [58]. Subsequent analyses demonstrated that primary MYC-high human SCLC tumors contained abundant guanosine nucleotides and that SCLC models with acquired chemoresistance exhibited increased MYC expression and IMPDH dependence [59]. Mechanistically, guanosine triphosphate (GTP) availability linked MYC-driven nucleotide synthesis to RNA polymerase I-dependent ribosome biogenesis [59]. Stable-isotope labeling in cultured SCLC cells further traced glucose- and glutamine-derived atoms into purine nucleotides, although these experiments did not quantify absolute pathway fluxes in vivo [59]. Collectively, these studies establish metabolic heterogeneity within SCLC and nominate arginine metabolism and IMPDH-dependent GTP synthesis as subtype-associated therapeutic vulnerabilities; however, these findings remain predominantly preclinical and require prospective clinical validation.

Whether transcription-factor subtype can predict arginine or guanosine nucleotide dependence is therefore a concrete open question, and current evidence suggests a partial rather than one-to-one correspondence. MYC drives progression of SCLC to a variant neuroendocrine subtype [9] and, in genetically engineered mice, a temporal shift from ASCL1-positive to NEUROD1-positive and then YAP1-positive states, while separately promoting POU2F3-positive tumors from a distinct cell of origin [62]. The MYC-high, ASCL1-low tumors in which arginine and IMPDH dependence were described [31,58,59] are therefore expected to be enriched within SCLC-N, but MYC activity is not confined to that subtype. Subtype assignment in patients is also graded rather than absolute: in an immunohistochemical series of 174 tumors, 69% were ASCL1-dominant and 17% NEUROD1-dominant, whereas POU2F3 expression (7%) was mutually exclusive with these factors [63]. Clinical data illustrate the consequence of selecting on enzyme deficiency alone: in a phase II trial of pegargiminase in 22 patients with relapsed ASS1-deficient SCLC, the best response was stable disease in four (18.2%); the trial closed early, and the investigators suggested that molecular stratification including MYC status may offer new opportunities [64]. A testable research direction follows. Subtype-annotated patient tumors and patient-derived models should be profiled jointly for ASCL1, NEUROD1, POU2F3, YAP1, and MYC status; for ASS1 and IMPDH1/2 expression and guanosine nucleotide pools; and for functional sensitivity to arginine deprivation and IMPDH inhibition, with isotope tracing to determine whether arginine uptake and de novo guanosine nucleotide synthesis differ by subtype. If subtype or MYC status predicts these dependencies, it could serve as a selection biomarker in the way that ASS1 deficiency has in pleural mesothelioma (Section 10).

### 5.4. Esophageal Carcinoma

Esophageal carcinoma has been studied considerably less than lung cancer from a metabolic standpoint, and its two dominant histologies differ in ways likely to be metabolically consequential. ESCC arises on a background of chronic mucosal injury and shares squamous lineage features with LUSC, whereas EAC develops through the metaplasia–dysplasia–carcinoma sequence of Barrett esophagus in the setting of chronic gastroesophageal reflux, obesity, and sustained exposure to bile acids at low pH [13]. Whether ESCC also shares the glycolytic emphasis of LUSC has not been tested directly. These distinct etiologies imply different metabolic selective pressures, and the bile-acid-associated metabolic subtype identified in lung adenocarcinoma (Section 7.1) has an obvious counterpart hypothesis in the esophagus; however, we are not aware of direct comparative flux measurements between ESCC and EAC, and the metabolic consequences of chronic bile-acid exposure in Barrett epithelium remain largely uncharacterized in patients.

A substantial metabolic dataset in esophageal cancer derives from spatially resolved profiling. Airflow-assisted desorption electrospray ionization mass spectrometry imaging applied to tissue from 256 patients with ESCC identified dysregulation of proline biosynthesis, glutamine metabolism, uridine metabolism, histidine metabolism, fatty-acid biosynthesis, and polyamine biosynthesis, and nominated the corresponding enzymes as spatially localized candidate targets, among them pyrroline-5-carboxylate reductase 2 (PYCR2), glutaminase (GLS), and uridine phosphorylase 1 (UPP1) [65]. Because these were tissue-level associations without isotope tracing or functional perturbation in patients, they identify candidate pathways rather than validated dependencies. Immune and stromal remodeling of the esophageal TME has separately been implicated in resistance to neoadjuvant immunochemotherapy (Section 4) [14] and metabolic contributions have been proposed [5]. Biofluid-based metabolic biomarkers in esophageal cancer nevertheless remain markedly less developed than in lung cancer, and, to our knowledge, no esophageal metabolic signature has undergone independent prospective validation.

### 5.5. Comparative Evidence and Evidence Gaps Across Thoracic Histologies

A central aim of this review is to separate metabolic changes that are shared across thoracic malignancies from those that constitute lineage- or genotype-specific dependencies, because only the latter are candidate therapeutic targets. Elevated aerobic glycolysis, lactate production, and extracellular acidification are common to most of these tumors and to many solid cancers generally; on their own, they mark a shared malignant phenotype rather than a selective vulnerability [15,17]. In contrast, several dependencies are lineage- or genotype-defined: ASS1-loss–driven arginine auxotrophy in non-epithelioid pleural mesothelioma [66], the MTAP-deletion/PRMT5 synthetic-lethal state in NSCLC [35], and arginine- and guanosine-nucleotide dependence in MYC-high small cell lung cancer [31,58,59]. Of these dependencies, those in mesothelioma and in MTAP-deleted tumors have been tested in biomarker-selected therapy (Section 10), whereas the glycolytic phenotype shared across histologies has not produced clinical benefit when targeted in unselected populations.

The quantity and maturity of evidence also differ markedly across the four disease groups and should be weighed accordingly. Lung cancer is supported by in vivo isotope-tracing studies in patients [24,25,26], large proteogenomic datasets [7,30], and biomarker cohorts with independent validation [67,68]. Esophageal cancer has substantial tissue-level and multi-omic data, including spatially resolved and multicenter metabolomic studies [47,65,69,70], but no prospectively validated biofluid signature. For pleural mesothelioma and thymic epithelial tumors, the evidence remains confined to small tissue cohorts, patient-derived xenografts, and single-center biomarker studies, none of which, to our knowledge, included more than 34 patients [10,12,71,72]. With the important exception of the ATOMIC-Meso trial of arginine deprivation (Section 10) [66], findings in these rare tumors should be regarded as hypothesis-generating rather than clinically established. Direct cross-tumor comparison is further constrained because studies differ in platform, specimen, and analytical pipeline, so apparent differences between diseases may partly reflect methodology rather than biology.

## 6. Analytical Platforms and Computational Workflows

### 6.1. Nuclear Magnetic Resonance (NMR) Spectroscopy

NMR spectroscopy is reproducible, non-destructive, and inherently quantitative, and because signal generation does not depend on ionization, it avoids the ion-suppression effects encountered in mass spectrometry. It can be applied to biofluids and tissue extracts, and high-resolution magic-angle spinning NMR allows analysis of intact tissue [16,73]. Its main limitation is sensitivity: even with high-field magnets, cryogenic probes, and hyperpolarization, detection limits are typically in the low-micromolar range, one to two orders of magnitude worse than for mass spectrometry, and low-abundance metabolites may go undetected [15,16,73].

### 6.2. Mass Spectrometry (MS)

Mass spectrometry, usually coupled to gas or liquid chromatography, offers greater sensitivity and broader metabolite coverage than NMR and has become the dominant platform in oncology research. Gas chromatography–mass spectrometry (GC–MS) suits volatile, thermally stable analytes such as amino acids, organic acids, and glycolytic intermediates after chemical derivatization, whereas liquid chromatography–tandem mass spectrometry (LC–MS/MS) separates polar, thermally labile, and structurally complex molecules, including sugar phosphates, nucleotides, and lipids, without derivatization. Untargeted profiling on high-resolution instruments, such as quadrupole time-of-flight (Q-TOF), Orbitrap, or Fourier transform ion cyclotron resonance (FTICR) analyzers, captures thousands of features for discovery using data-dependent (DDA) or data-independent (DIA) acquisition, but is limited by incomplete annotation and difficult interpretation. Targeted analysis on triple-quadrupole instruments in multiple reaction monitoring (MRM) mode, with isotopically labeled internal standards, quantifies predefined panels of tens to several hundred metabolites with the precision required for clinical translation [16].

### 6.3. Bioinformatics Pipelines and Public Repositories

Raw spectra are converted into feature tables through noise reduction, baseline correction, retention-time alignment, and peak picking, using software such as XCMS, MZmine, MS-DIAL, or OpenMS, followed by exploratory principal component analysis (PCA) and supervised methods such as partial least squares–discriminant analysis (PLS-DA) [16,21,74]. Annotated features are mapped onto pathways using resources such as the Kyoto Encyclopedia of Genes and Genomes (KEGG), and integrated platforms such as MetaboAnalyst support normalization, batch-effect correction, statistical analysis, and pathway enrichment [16,74]. Metabolite identification remains the main bottleneck, because accurate-mass and tandem mass spectrometry (MS/MS) spectra must be matched against repositories that differ in coverage and annotation quality. Annotations are therefore reported at standardized confidence levels. A widely used five-level scheme for high-resolution MS runs from an exact mass of interest (Level 5) and an unequivocal molecular formula (Level 4), through tentative candidates (Level 3) and probable structures supported by library spectral matches (Level 2), to Level 1 confirmation against an authentic standard analyzed under the same conditions [21,75]. The statistical safeguards that these analyses require are discussed in Section 6.5.

### 6.4. Spatial Metabolomics and In Vivo Metabolic Imaging

The platforms described above measure metabolites in bulk tissue extracts and therefore average across malignant, stromal, and immune compartments, the same compartments whose metabolic interactions Section 4 describes. Mass spectrometry imaging addresses this limitation by acquiring spectra across tissue sections while preserving spatial coordinates, most commonly using matrix-assisted laser desorption/ionization (MALDI) or desorption electrospray ionization (DESI) sources. Applied to 330 resected LUSC specimens, high-mass-resolution imaging mass spectrometry defined four tumor-specific and four stroma-specific metabolic subtypes, one tumor subtype being independently prognostic with respect to stage and one stromal subtype associated with chemotherapy resistance in an independent neoadjuvant-treated cohort [76]. Spatially resolved profiling has been applied comparably in ESCC (Section 5.4) [65]. These approaches deliver compartment-level resolution that bulk metabolomics cannot, although they remain semi-quantitative, cover fewer metabolites than chromatography-coupled platforms, and do not by themselves report isotope-resolved flux.

In vivo metabolic measurement in patients currently rests principally on positron emission tomography. ^18^F-fluorodeoxyglucose (FDG) PET is embedded in routine thoracic staging and response assessment and remains the only metabolic imaging assay in routine clinical use in thoracic oncology; any proposed metabolomic biomarker must therefore demonstrate value beyond it rather than merely correlate with it, a comparison that the biofluid studies discussed in Section 7 have generally not made. Tracers reporting other pathways have reached exploratory clinical evaluation, including (4S)-4-(3-[^18^F]fluoropropyl)-L-glutamate for system xc− transport in NSCLC [77]. Separately, hyperpolarized ^13^C magnetic resonance using dissolution dynamic nuclear polarization permits real-time non-invasive measurement of apparent pyruvate-to-lactate conversion and has been demonstrated in patients, initially in prostate cancer [78]. The signal reflects label exchange shaped by substrate delivery, membrane transport, LDH activity, and the endogenous lactate pool rather than net glycolytic flux, so the approach narrows rather than closes the abundance-versus-flux gap emphasized throughout this review. Thoracic application is further constrained by respiratory and cardiac motion, low tissue density, the short lifetime of the hyperpolarized signal, and air–tissue susceptibility artifact, and we are not aware of published studies in human thoracic tumors.

### 6.5. Statistical and Computational Considerations

Because metabolomic datasets are high-dimensional and cancer cohorts, especially in the rare thoracic tumors, are small, statistical and computational rigor is central to credible biomarker development. The number of measured features commonly exceeds the number of patients, so models are vulnerable to overfitting and to spurious associations unless multiple-testing correction, principled feature selection, and strict separation of training and test data are enforced; information from the test set can also influence feature selection or normalization, a form of leakage that overstates performance [79]. Several biomarker studies discussed in this review report discrimination metrics derived within the same dataset used to build the model, or in internal splits only, which inflates apparent performance, as the implausibly extreme hazard ratios noted in Section 7.2 illustrate. Normalization, batch-effect correction, and missing-data handling further shape results and should be prespecified; integrated platforms such as MetaboAnalyst provide standardized implementations [74], and mQACC guidance addresses analytical quality control [80]. For predictive and machine-learning models, reporting frameworks such as TRIPOD+AI support transparency and reproducibility [81]; external validation remains necessary for clinical transportability. Computational reproducibility requires the same discipline as the laboratory work: analysis code, software and library versions, parameter settings, and the random seeds used for data splitting and model fitting should be recorded and deposited alongside the data, since identical raw spectra analyzed with different pipeline versions can yield different feature tables and different models. These safeguards are prerequisites for any metabolomic signature intended to inform clinical decisions and are summarized among the translational barriers in Section 11.

## 7. Translational Applications and Clinical Utility

The translational potential of metabolomics in thoracic oncology is currently most evident in biomarker discovery and minimally invasive disease detection, whereas its use for routine drug selection and patient stratification remains investigational [15,16,20]. Biofluid studies nevertheless demonstrate the feasibility of detecting lung-cancer-associated metabolic alterations across several clinically accessible matrices. Serum, urine, bronchoalveolar lavage fluid and plasma have each yielded lung-cancer-associated metabolic signatures using gas chromatography–mass spectrometry (GC–MS) and liquid chromatography–mass spectrometry (LC–MS); the principal studies are described in Section 7.1 and summarized in Table 2 [67,68,82]. Their shared limitation is that all are retrospective case–control designs rather than prospective screening cohorts, so their performance in the population where such an assay would be deployed remains unknown.

However, the biological responsiveness that makes circulating metabolomics attractive for precision oncology also complicates its clinical interpretation. Plasma and serum metabolomes integrate tumor-associated signals with metabolism in the liver, skeletal muscle, and other organs, as well as diet and fasting status, microbiome activity, physical activity, comorbidities, and medication or treatment exposure. A circulating metabolite profile therefore cannot be assumed to provide a tumor-specific readout or a direct measure of pathway activity within the primary tumor [15,18]. Translation of such profiles from exploratory associations into clinically reliable assays requires prospective control of patient-related, pre-analytical, analytical, and post-analytical sources of variation [15,18,20].

Pre-analytical control is particularly important because cellular and enzymatic processes continue in unseparated blood after venipuncture. Delayed separation of plasma or serum from blood cells can reduce glucose and increase lactate through continued erythrocyte glycolysis while perturbing numerous additional analytes [18,86,87]. Hemolysis can further distort circulating profiles by releasing intracellular metabolites, whereas contamination with residual cellular material had comparatively small effects [86]. Following plasma or serum separation, metabolite stability depends on the analyte, analytical platform, storage duration and temperature, and thawing conditions. One or two controlled freeze–thaw cycles often have limited effects, whereas repeated or poorly controlled cycles can alter subsets of metabolites [18].

Accordingly, collection and processing protocols should prespecify the collection tube and anticoagulant, relevant fasting and collection-time conditions, maximum permissible delay and temperature before centrifugation, centrifugation conditions, aliquot volume, storage temperature and duration, and number of freeze–thaw cycles (Table 3). ISO 23118:2021 provides formal requirements and recommendations for the pre-examination handling of urine, venous blood plasma, and serum, while the metabolomics Quality Assurance and Quality Control Consortium (mQACC) recommendations provide a framework for analytical quality assurance, quality control, and reporting [80,88]. For long-term blood-metabolomics studies, prompt separation and aliquoting followed by validated low-temperature storage, commonly at −80 °C, is preferable to an unqualified requirement for “immediate freezing,” because acceptable conditions may be analyte- and platform-dependent [18,88]. Analytical workflows commonly incorporate randomized or appropriately balanced sample order, system-suitability testing, analytical blanks, internal standards where applicable, and pooled quality-control samples distributed throughout each batch with predefined acceptance criteria, and mQACC recommends reporting each of these [80].

Finally, signals detected by untargeted metabolomics should remain classified as annotated features until their identities are supported at a clearly stated confidence level. Definitive metabolite identification generally requires comparison with an authentic standard analyzed under identical conditions using at least two independent and orthogonal properties [16,21,89]. Candidate biomarkers should subsequently be transferred to fit-for-purpose quantitative assays and evaluated in independent, preferably prospective cohorts, with demonstration of clinical validity and added value before clinical deployment [15,20].

### 7.1. Early Screening Diagnostics and Liquid Biopsies

Despite these barriers, metabolomics and related metabolic analyses have shown potential to identify candidate diagnostic and treatment-response biomarkers in thoracic oncology, although their routine clinical implementation remains limited [15,20,67,68,83]. Stable-isotope tracing can additionally provide mechanistic information that is not obtainable from steady-state biomarker profiling. In patients with resectable NSCLC, intravenous [U-^13^C]lactate produced labeling of tumor TCA-cycle metabolites, demonstrating that at least some human NSCLCs use carbon derived from circulating lactate in vivo [24]. These human experiments established lactate utilization but did not quantitatively demonstrate that lactate contributes more TCA-cycle carbon than glucose. The direct comparison indicating a greater contribution from lactate than glucose was performed in mouse NSCLC xenografts using concurrent isotope tracers [24], consistent with systemic murine tracer studies showing that circulating lactate is a principal direct TCA-cycle substrate in multiple tissues and tumors [29]. Histology-associated metabolic differences have also been identified. Human LUSC specimens showed higher GLUT1 expression and ^18^F-FDG uptake than LUAD. Selective sensitivity of squamous tumors to GLUT1 depletion and pharmacological glycolytic inhibition was demonstrated in cell lines and in xenografts derived from them. The patient-derived xenograft and genetically engineered mouse models in that study documented the GLUT1-expression difference rather than sensitivity to inhibition [6].

Low-dose computed tomography (LDCT) remains the established screening modality for eligible high-risk populations and reduces lung-cancer mortality; however, screening can generate false-positive findings, indeterminate pulmonary nodules, incidental findings, and cumulative radiation exposure [91]. Metabolite-based liquid biopsies should therefore be investigated as potential adjuncts for nodule risk stratification rather than as replacements for LDCT. In a small exploratory human study, GC–MS was applied to serum and urine from 32 patients with lung cancer and 29 healthy controls, together with bronchoalveolar lavage fluid from 24 patients with lung cancer and 30 patients with nonmalignant pulmonary diseases [82]. Altered metabolites were identified in each matrix, particularly within amino acid-associated pathways, but only four alterations were shared across serum, urine, and bronchoalveolar lavage fluid, illustrating both the feasibility and the matrix dependence of multi-biofluid profiling [82].

More extensive human case–control studies provide additional evidence of biomarker potential. LC–MS analysis of urine from 469 patients with lung cancer and 536 population controls, followed by confirmation in an independent cohort of 80 cases and 78 controls, identified creatine riboside and N-acetylneuraminic acid as candidate diagnostic and prognostic markers [68]. Both metabolites were also enriched in human lung tumors relative to adjacent nonmalignant tissue, strengthening their association with tumor biology [68]. More recently, targeted quantitative analysis of 680 archived plasma samples and an independent validation set of 216 samples produced a 10-metabolite NSCLC model [67]. After incorporation of smoking history, validation-set areas under the receiver-operating characteristic curve were approximately 0.92 for stage I–II disease and 0.89 for stage I disease [67]. Nevertheless, these were retrospective case–control studies rather than prospective LDCT-screening cohorts; their performance for evaluating screen-detected nodules and their added clinical value beyond established risk models remain unresolved.

Targeted LC–MS analysis of resected human lesions from 181 patients spanning atypical adenomatous hyperplasia (AAH), adenocarcinoma in situ (AIS), minimally invasive adenocarcinoma (MIA), and invasive adenocarcinoma (IAC) identified progressively altered metabolic patterns across these histologic categories [84]. A separate human plasma cohort comprising 10 patients with benign pulmonary disease, 32 with AIS, 22 with MIA, and 28 with IAC generated candidate metabolite panels that distinguished early LUAD from benign disease [84]. Because the tissue and plasma cohorts were separate and cross-sectional, these results support stage-associated metabolic differences but do not establish longitudinal metabolic evolution within individual patients or completely exclude systemic confounding.

Within the human IAC tissue cohort, unsupervised metabolomic clustering identified three metabolic subtypes, of which subtype III showed the highest bile-acid metabolism; subtypes II and III were both associated with adverse clinicopathologic features and poorer disease-free survival [84]. Functional evidence was preclinical: selected bile acids promoted migration in H1299 lung adenocarcinoma cells, while cholic acid and glycochenodeoxycholic acid increased pulmonary colonization after tail-vein injection of A549 cells into athymic mice [84]. These findings nominate bile-acid–TGR5 signaling as a candidate metabolic vulnerability but do not establish it as a validated therapeutic target in patients. Separately, early-on-treatment serum profiling in patients with advanced NSCLC identified hypoxanthine and histidine as candidate predictors of response to PD-1 blockade [83]; these results remain investigational and require larger prospective validation.

### 7.2. Treatment Monitoring and Immunotherapy Response

Longitudinal tracking of the cancer metabolome offers a minimally invasive approach to evaluating therapeutic response, predicting resistance, and stratifying risk across thoracic and other advanced solid tumors. In patients with advanced NSCLC receiving PD-1 blockade, mass-spectrometry profiling of serum collected 2–3 weeks after the first treatment identified a two-metabolite panel comprising hypoxanthine and histidine in a discovery cohort of 43 patients receiving second-line or third-line nivolumab, with subsequent evaluation in two small sets of 21 and 10 patients [83]. Higher early on-treatment levels of both metabolites distinguished responders from non-responders and were associated with improved progression-free survival (PFS) and overall survival (OS) in the discovery cohort [83]. Two features materially limit interpretation. The larger evaluation set received a different agent, tislelizumab, as first-line combination therapy rather than second-line or third-line nivolumab, and therefore does not reproduce the discovery setting; and the reported hazard ratios for PFS, on the order of 0.08 to 0.14, correspond to effect sizes far larger than cohorts of this size can credibly establish and are, in our assessment, likely inflated in the discovery cohort, in which the panel was both selected and evaluated in the same small dataset. Because serum collection preceded the first routine radiographic response assessment, performed every 8 weeks, this panel may facilitate earlier identification of patients likely to benefit from continued PD-1 blockade, although adequately powered prospective validation remains necessary before any clinical inference is drawn [83]. Mechanistically, hypoxanthine may reflect altered adenosine catabolism, whereas greater histidine availability may support antitumor immune-cell function; however, these interpretations remain preliminary, and the signature should not yet be considered evidence of restored systemic metabolic homeostasis [83]. Tumor use of circulating lactate and of lipid synthesis may also support survival under therapeutic stress, although neither has been measured during treatment in patients [24]. Circulating metabolic measurements, potentially integrated with established markers such as serum lactate dehydrogenase, may therefore provide complementary endpoints for immunotherapy-monitoring protocols [92].

Evidence from other tumor types further demonstrates the value of longitudinal metabolomic monitoring during immunotherapy. In patients with advanced melanoma or metastatic renal cell carcinoma treated with nivolumab, serial serum metabolomics identified an on-treatment increase in the kynurenine-to-tryptophan ratio as a marker of adaptive resistance associated with shorter OS [93]. In melanoma, increases measured 4–6 weeks after treatment initiation were associated with an increased risk of death, while comparable associations were independently observed in nivolumab-treated renal cell carcinoma [93]. Kynurenine is generated through indoleamine 2,3-dioxygenase (IDO)- or tryptophan 2,3-dioxygenase (TDO)-mediated tryptophan catabolism and can suppress T-cell proliferation. Moreover, the on-treatment kynurenine-to-tryptophan ratio correlated with programmed death-ligand 1 (PD-L1) and IDO1 expression in melanoma specimens, consistent with an adaptive immunosuppressive program that counterbalances checkpoint inhibition [93]. These findings suggest the value of dynamic metabolomic monitoring for patient stratification and provide a rationale for biomarker-guided trials combining PD-1 blockade with interventions targeting kynurenine production or signaling [93].

## 8. Rare Thoracic Tumors

Rare thoracic tumors illustrate both the potential and the current limitations of metabolic profiling, because the available evidence is derived largely from small tissue cohorts and experimental models rather than clinically validated assays [10,12]. In TETs, Alwahsh and colleagues applied high-resolution magic-angle-spinning proton nuclear magnetic resonance spectroscopy (HRMAS ^1^H-NMR) to 15 snap-frozen tumors, 12 thymomas and 3 thymic carcinomas, and four non-neoplastic thymus samples, detecting 37 metabolites across the cohort [10]. The investigators classified type A, AB, and B1 thymomas as clinically indolent and grouped type B2 and B3 thymomas with thymic carcinomas as aggressive; within this exploratory dataset, metabolite profiles separated these two prespecified groups [3,10]. The aggressive group exhibited higher lactate, glutathione, alanine, inosine, and oxypurinol, whereas cysteine and myo-inositol were higher in the indolent group [10]. Metabolite-based pathway analysis, complemented by transcriptomic enrichment analysis in an independent TET dataset, implicated glycolysis, glutamine-related metabolism, and glutathione-dependent redox regulation [10].

These findings should nevertheless be interpreted cautiously. Steady-state metabolite concentrations do not establish increased glutaminolytic or TCA-cycle flux, and the study did not test metabolic inhibitors or validate its classification results in an independent metabolomic cohort [10,27]. Accordingly, glycolysis, glutamine metabolism, and redox homeostasis represent candidate pathways for functional investigation rather than established therapeutic vulnerabilities. Complementary human evidence has subsequently emerged from a prospective surgical study of 20 patients with suspected thymoma, comprising 9 patients with thymoma and 11 with benign thymic cysts [71]. Mass-spectrometry-based metabolomics distinguished thymomas from adjacent thymus and benign lesions, while intraoperative ^13^C-glucose infusion in 11 participants revealed differences in central-carbon labeling patterns [71]. These results are consistent with altered glucose, lactate, pyrimidine, and glycerophospholipid metabolism in thymoma; however, isotope enrichment does not itself provide absolute pathway-flux measurements, and the small cohort requires independent validation [27,71].

In PM, Chen and colleagues investigated the feasibility of establishing PDX models from ultrasound-guided pleural biopsies [12]. Five pathologically confirmed PM specimens were used for attempted engraftment, of which two, one epithelioid and one sarcomatoid tumor, successfully generated serially transplantable PDX models that retained key histopathologic characteristics of the corresponding patient tumors [12]. No patient serum was analyzed. Untargeted GC–MS was performed on serum collected from immunodeficient mice bearing the two human PM xenografts and from tumor-free control mice [12]. Kynurenine, uridine, uric acid, and inosine were elevated in the serum of mice bearing both PDX models; however, these circulating concentrations reflect the combined metabolism of the human xenograft and the murine host [12]. Only the first PDX model separated from controls by unsupervised principal component analysis; the second did not separate, and its separation by partial least-squares discriminant analysis was ambiguous [12]. These findings therefore cannot be interpreted as a human diagnostic signature or as evidence that the identified metabolites are validated therapeutic targets. Instead, they provide hypothesis-generating evidence that tryptophan and nucleotide metabolism warrant investigation in larger model panels and directly collected patient specimens.

Human circulating metabolomics evidence in PM remains preliminary. In a separate study, targeted LC–MS/MS and flow-injection MS/MS (Biocrates p180) were used to analyze serum obtained before and after radical hemithoracic radiotherapy from 19 of 34 enrolled patients with PM [72]. Treatment was associated with changes in amino acids, acylcarnitines, and phosphatidylcholines, with arginine metabolism and polyamine biosynthesis among the most perturbed pathways [72]. Because this was a small, single-center exploratory cohort and the circulating profiles captured systemic responses to radiotherapy rather than tumor metabolism alone, the reported associations require prospective validation before they can be considered prognostic biomarkers. These studies show that metabolic investigation of rare thoracic tumors is feasible. They also show what it still needs: larger multicenter cohorts, direct analysis of patient-derived specimens, functional pathway validation, and a clear separation of tumor-intrinsic metabolism from host systemic responses.

## 9. Precision Oncology and Multi-Omics Integration

Integrating metabolomics with genomic, transcriptomic, proteomic, single-cell, spatial, microbiome, and clinical data is particularly important in thoracic oncology, where metabolic phenotypes arise from interactions among oncogenic signaling, lineage, stromal and immune composition, and treatment exposure [15]. Gene expression and steady-state metabolite profiles each give only a partial view of pathway activity in vivo, so identifying causal relationships between levels of regulation requires integrated designs [16,25]. Integration may combine datasets that share a common regulatory network, or connect clinical data with parallel omic and microbiome layers [15]; proteogenomic studies of LUSC and LUAD illustrate the approach, linking copy-number alterations and somatic mutations to protein, phosphoprotein, and acetylprotein features, including post-translational regulation of glycolytic enzymes in LUSC [7,30].

The value of these approaches lies in their combination. Flux measurement (Section 2), immunometabolic remodeling of the tumor microenvironment (Section 4), and histology-specific dependencies (Section 5) are each informative in isolation, but are likely to become predictive only when integrated within a common computational architecture: isotope-resolved flux supplies the functional axis that static proteogenomics cannot, single-cell and spatial profiling localize that flux to defined stromal and immune compartments, and joint modeling links both to genotype and clinical outcome. The methodological claim is therefore specific: no single layer isolates causal, actionable dependencies, whereas their combination can. Pairing in vivo ^13^C-tracing with single-cell mapping of the ESCC microenvironment would, for instance, separate tumor-intrinsic metabolic vulnerability from microenvironment-driven resistance, a distinction neither method resolves alone. To our knowledge, such a study has not been reported; identifying this as an unfilled gap is a concrete near-term objective rather than a description of current practice.

Realizing this integration requires sampling designs that can attribute metabolic signals to their cellular source. Bulk tissue and biofluid measurements average across malignant, immune, and stromal compartments, yet these compartments partition nutrients differently. In murine tumors, myeloid cells take up the most glucose whereas malignant cells take up the most glutamine [38], so compartment-resolved approaches are essential rather than optional. Paired tumor-tissue and blood sampling distinguishes tumor-derived from systemic signals; spatially resolved metabolomics [65,76] and single-cell profiling [14] assign metabolic and immune states to defined tissue compartments and cell populations; and in vivo isotope tracing assigns pathway activity rather than abundance. Equally important is dynamic sampling before, during, and after treatment, which separates intrinsic tumor metabolic change from the host systemic response to therapy and distinguishes transient from sustained shifts. Longitudinal, compartment-aware designs of this kind, rather than additional single-timepoint bulk profiling, are the logical next step for the field, and would, for example, allow the tumor-intrinsic and microenvironmental contributions discussed in Section 4 and Section 5 to be disentangled in the same patients.

## 10. Metabolism-Targeted Therapeutics

Clinical translation of metabolism-targeted therapies has been uneven, and the strength of evidence differs substantially among agents. The glycolysis-disrupting glucose analog 2-deoxy-D-glucose (2-DG) was evaluated with weekly docetaxel in a 34-patient phase I study of advanced solid tumors. The study established 63 mg/kg/day as a clinically tolerable dose, but the best responses comprised one partial response and 11 cases of stable disease. Target engagement was assessed directly: of 17 patients who underwent ^18^F-FDG PET at baseline and again after one week of 2-DG alone, only five showed a fall in summed standardized uptake value, by 11% to 49% [94]. As a nonrandomized dose-escalation study, it therefore established feasibility and dosing rather than definitive efficacy, and the pharmacodynamic data indicate that the intended metabolic effect was achieved in a minority of those assessed [94]. PFK-158, a compound developed as a PFKFB3-directed glycolysis inhibitor, subsequently entered first-in-human phase I testing in patients with advanced solid malignancies (NCT02044861). To our knowledge, no mature, peer-reviewed efficacy analysis of PFK-158 has been published in this population, so the published literature does not support the specific assertion that PFK-158 was discontinued for insufficient efficacy. The registry record lists the trial status as unknown, was last updated in 2015, and has no posted results.

Glutaminase inhibition with telaglenastat (CB-839) provides more definitive randomized evidence. Outside thoracic oncology, the CANTATA trial randomized 444 patients with advanced clear-cell renal cell carcinoma to telaglenastat plus cabozantinib or placebo plus cabozantinib. Telaglenastat did not improve the primary endpoint of PFS, with median values of 9.2 and 9.3 months, respectively (hazard ratio, 0.94; 95% confidence interval, 0.74–1.21; *p* = 0.65) [95]. In thoracic oncology, the biomarker-selected KEAPSAKE phase II trial translated preclinical evidence linking KEAP1 loss and NRF2-pathway activation to glutamine dependence into a randomized evaluation of telaglenastat or placebo combined with pembrolizumab, carboplatin, and pemetrexed in first-line metastatic KEAP1/NRF2-mutated nonsquamous NSCLC [28]. The trial (NCT04265534) had randomized 40 patients when it was unblinded and was then terminated; the registry gives lack of clinical benefit as the reason, but no results have been posted, and no peer-reviewed efficacy report has been published to date. The KEAPSAKE outcome should therefore be treated as an early termination without a peer-reviewed efficacy publication, rather than as equivalent in evidentiary strength to the fully reported CANTATA result.

MCT1 inhibition has also reached clinical evaluation. In the first-in-human phase I study of AZD3965, 40 patients with advanced solid tumors or lymphoma received treatment during dose escalation. Drug exposures reached predicted target concentrations, pharmacodynamic measurements demonstrated target engagement, and 10 mg twice daily was selected as the recommended phase II dose for expansion in cancers generally characterized by high MCT1 and low MCT4 expression [96]. Dose-limiting toxicities were mostly dose-dependent, reversible, and asymptomatic ocular changes [96]. These findings establish pharmacologic feasibility and target engagement but do not constitute a registrational efficacy assessment.

In contrast to these unselected programs, arginine deprivation in a biomarker-defined population has produced what is, to our knowledge, the first positive randomized phase 2/3 result in a thoracic malignancy for an agent directed at a tumor-specific metabolic dependency, as distinct from the classical antimetabolites long used as cytotoxic chemotherapy. Tumors lacking argininosuccinate synthetase 1 (ASS1) cannot synthesize arginine de novo and are consequently auxotrophic for it. In the ATOMIC-Meso trial, 249 patients with non-epithelioid pleural mesothelioma were randomized to pegargiminase (pegylated arginine deiminase, ADI-PEG20) or placebo in combination with pemetrexed plus cisplatin or carboplatin [66]. Median overall survival was 9.3 months (95% CI, 7.9–11.8) with pegargiminase-chemotherapy versus 7.7 months (95% CI, 6.1–9.5) with placebo-chemotherapy (hazard ratio for death, 0.71; 95% CI, 0.55–0.93; *p* = 0.02), and median progression-free survival was 6.2 versus 5.6 months (hazard ratio, 0.65; 95% CI, 0.46–0.90; *p* = 0.02); grade 3 or higher treatment-emergent adverse events related to pegargiminase or placebo occurred in 28.8% and 16.9% of patients, respectively [66]. The absolute gain in median overall survival, 1.6 months, is modest, and the result applies only to non-epithelioid histology. The trial is nevertheless significant for a reason beyond its effect size: it directed an agent at a defined metabolic dependency in a population enriched for that dependency.

Eligibility was defined by non-epithelioid histology rather than by a direct assay of ASS1 status: immunohistochemical confirmation of ASS1 deficiency was not required for enrollment, which the investigators attribute to differences in antibody sensitivity between commercial and academic laboratories, and they list this as a limitation [66]. Histology was used as an enrichment proxy on evidence generated earlier in the same program. Non-epithelioid tumors show an approximately three-fold higher rate of ASS1 deficiency than epithelioid tumors, 60% versus 20% [66]. In the preceding randomized phase 2 ADAM trial, which enrolled only patients with immunohistochemistry-demonstrated ASS1-deficient tumors, pegargiminase prolonged median progression-free survival by 1.2 months compared with best supportive care [97]. The ATOMIC-Meso investigators describe that program as validating ASS1 as a prognostic and predictive biomarker [66]; as ADAM enrolled only ASS1-deficient tumors, however, it could not by itself show that tumors with intact ASS1 do not benefit.

Pharmacodynamic data confirm that the intended metabolic effect was achieved in patients: plasma arginine declined with a reciprocal rise in citrulline, and anti-pegargiminase antibodies were detected in 37 of 38 patients (97.4%) by week 25 [66]. Reported or proposed mechanisms of resistance include ASS1 re-expression through promoter demethylation, which permits recycling of citrulline to arginine, together with metabolic rewiring via polyamines, autophagy, and macrophage recruitment [66]. Arginine dependence has independently been identified in MYC-driven SCLC models (Section 5.3) [31], and arginine and polyamine metabolism were among the most perturbed pathways in circulating profiles from patients with pleural mesothelioma undergoing radiotherapy (Section 8) [72], although the radiotherapy-associated changes reflect a systemic response and do not by themselves indicate arginine dependence. Resistance has been better characterized for this program than for the others compared here: for 2-DG, PFK-158, telaglenastat, and the MTA-cooperative PRMT5 inhibitors, acquired resistance has not been described in treated patients, which probably reflects how few patients have been treated to progression with pharmacodynamic and molecular sampling rather than an absence of resistance.

A second biomarker-defined metabolic strategy has advanced into clinical testing in NSCLC. Homozygous MTAP deletion causes accumulation of methylthioadenosine, which partially inhibits PRMT5 and renders MTAP-deleted cells selectively dependent on residual PRMT5 activity [35]. MTA-cooperative PRMT5 inhibitors are designed to exploit this state. In the first-in-human study of AMG 193 (anvumetostat) in MTAP-deleted solid tumors, 80 patients were treated during dose exploration; among 42 efficacy-evaluable patients at active and tolerable dose levels, the objective response rate was 21.4% (95% CI, 10.3–36.8), with responses across eight tumor types including squamous and non-squamous NSCLC [36]. This program differs from the agents above in targeting a synthetic-lethal dependency created by a genomic deletion rather than inhibiting a rate-limiting enzyme within an active pathway, and in incorporating patient selection from first-in-human dosing onward. The strategy has now reached randomized testing: a phase 2/3 trial is comparing navlimetostat (MRTX1719/BMS-986504) plus pembrolizumab and chemotherapy with placebo plus pembrolizumab and chemotherapy in first-line metastatic NSCLC with homozygous MTAP deletion (NCT07063745), and phase I/II studies of the same agent (NCT05245500) and of TNG462 (NCT05732831) include patients with mesothelioma or NSCLC (Table 4).

Taken together, these programs support a more specific conclusion than a general pessimism about metabolic therapy, although the number of informative trials is small. Preclinical metabolic dependence, tolerability, and pharmacodynamic target engagement do not by themselves translate into clinical benefit, and the programs that failed, namely 2-DG, PFK-158, and telaglenastat in CANTATA, shared the feature that patients were enrolled without a demonstrated, tumor-specific metabolic dependency. Selection was not their only weakness: 2-DG lowered tumor FDG uptake in only 5 of 17 patients assessed [94], and the investigators of CANTATA list the absence of biomarker data bearing on the effect of either agent on glutamine and glucose metabolism among the trial’s limitations [95]. KEAPSAKE attempted stratification but used NRF2-pathway mutation as a proxy for glutaminase dependence, without confirming that dependence in individual tumors or measuring target engagement in vivo; the STK11 and KEAP1 literature discussed in Section 4 indicates that these genotypes alter immune as well as metabolic phenotype, so a mutation-defined population is not necessarily a metabolically homogeneous one [51,53,98]. By contrast, the arginine-deprivation program enriched for a dependency that earlier work had characterized. The randomized phase 2 ADAM trial enrolled only patients with ASS1-deficient tumors and showed longer progression-free survival with pegargiminase plus best supportive care than with best supportive care alone [97], and ATOMIC-Meso used non-epithelioid histology as a proxy for that dependency, with on-treatment pharmacodynamic confirmation [66]. The MTAP–PRMT5 program goes further, selecting on a genomic deletion that generates the dependency it targets and confirming intratumoral PRMT5 inhibition in paired biopsies [36,37]. The discriminating variable across this evidence is therefore not the plausibility of the metabolic target but whether the dependency was demonstrated, directly or through a validated proxy, in the patients actually enrolled, and whether target engagement was measured once they were treated. With only one positive randomized trial, this inference rests on few data and may be confounded by differences in drug potency, toxicity, and disease setting. Future development should accordingly pair molecular and metabolic patient selection with direct confirmation of target engagement, ideally including an imaging or isotope-based pharmacodynamic readout (Section 6.4), and with combinations designed to address compensatory metabolic pathways [98].

**Table 4:** Metabolism-targeted therapeutic strategies relevant to thoracic oncology, compared by target and rationale, patient-selection biomarker, clinical setting, efficacy, principal toxicity and pharmacodynamic evidence where reported, and evidence status. The discriminating feature of the positive programs is selection on a demonstrated dependency, directly or through a validated proxy, rather than the plausibility of the target. Registry statuses were checked on ClinicalTrials.gov on 10 October 2026. 1L, first line; AE, adverse event; ctDNA, circulating tumor DNA; DCR, disease-control rate; FDG, fluorodeoxyglucose; HR, hazard ratio; IHC, immunohistochemistry; ORR, objective response rate; OS, overall survival; PET, positron emission tomography; PFS, progression-free survival; PR, partial response; RCC, renal cell carcinoma; RCT, randomized controlled trial; RP2D, recommended phase II dose; SD, stable disease; SDMA, symmetric dimethylarginine; SUV, standardized uptake value.

Agent/Target	Rationale	Selection Biomarker	Setting/Trial [Ref]	Efficacy	Key Toxicity; Pharmacodynamic Evidence	Evidence Status (Section 1)/Interpretation
2-DG/glycolysis	Glucose analog inhibits glycolysis	None	Phase I + docetaxel, advanced solid tumors [94]	1 PR, 11 SD; 63 mg/kg/day tolerable	Reversible hyperglycemia; grade 3 QTc prolongation 22% at 63–88 mg/kg; FDG-PET after one week of 2-DG alone in 17 patients, with a fall in summed SUV in 5 (11–49%)	Insufficiently supported: feasibility and dosing only; unselected.
PFK-158/PFKFB3	Glycolysis inhibition	None	Phase I, advanced solid tumors (NCT02044861)	No peer-reviewed efficacy report; registry status unknown, not updated since 2015, no results posted	Not reported	Insufficiently supported: no efficacy data; unselected
Telaglenastat/glutaminase	Block glutaminolysis	None	CANTATA randomized phase II, RCC + cabozantinib [95]	No PFS benefit (9.2 vs. 9.3 mo; HR 0.94)	As per combination; no biomarker data on glutamine or glucose metabolism (stated limitation)	Insufficiently supported: negative RCT; unselected.
Telaglenastat/glutaminase	KEAP1/NRF2 → glutamine dependence [28]	KEAP1/NRF2 mutation (proxy)	KEAPSAKE randomized phase II, 1L nonsquamous NSCLC (NCT04265534)	Terminated; registry reason “lack of clinical benefit” (*n* = 40); no results or peer-reviewed report	Not reported	Insufficiently supported: terminated for lack of benefit; mutation used as proxy for dependence.
AZD3965/MCT1	Inhibit MCT1-mediated lactate transport (influx and efflux)	High MCT1/low MCT4 (expansion)	Phase I, solid tumors/lymphoma [96]	Target engagement; RP2D 10 mg twice daily	Dose-limiting reversible ocular changes; troponin rise, acidosis; target engagement shown	Insufficiently supported for efficacy: target engagement shown
Pegargiminase (ADI-PEG20)/arginine deprivation	ASS1-deficiency arginine auxotrophy	Non-epithelioid histology as a proxy (ASS1 loss in 60% of non-epithelioid vs. 20% of epithelioid tumors); ASS1 IHC not required for enrollment	ATOMIC-Meso phase 2/3, non-epithelioid PM + pemetrexed and cisplatin or carboplatin [66]	OS 9.3 vs. 7.7 mo (HR 0.71); PFS 6.2 vs. 5.6 mo (HR 0.65)	Grade ≥ 3 pegargiminase/placebo-related AEs 28.8% vs. 16.9%; plasma arginine fell with reciprocal citrulline rise; anti-pegargiminase antibodies in 37/38 (97.4%) by week 25; resistance via ASS1 re-expression	Clinically supported: positive RCT in non-epithelioid PM, a population enriched for ASS1 loss
Pegargiminase/arginine deprivation	Arginine auxotrophy	ASS1 deficiency (<5% IHC)	Phase II, relapsed SCLC [64]	SD in 4/22 (18.2%); closed early	Not detailed here	Insufficiently supported: no objective responses; MYC stratification proposed.
Pegargiminase/arginine deprivation	Arginine auxotrophy	ASS1-deficient non-squamous NSCLC	Phase I + pemetrexed/cisplatin [99]	PR 47.6%; DCR 85.7%; mPFS 4.2, mOS 7.2 mo	Not detailed here	Promising but preliminary: single-arm, biomarker-selected
AMG 193 (anvumetostat)/MTA-cooperative PRMT5	MTAP-deletion synthetic lethality	MTAP deletion	Phase I/II MTAPESTRY 101 (NCT05094336; active, not recruiting) [36]	ORR 21.4% (42 evaluable), incl. NSCLC	Nausea 48.8%, fatigue 31.3%, vomiting 30.0%; complete intratumoral PRMT5 inhibition in paired biopsies at ≥480 mg; ctDNA clearance	Promising but preliminary: phase I activity; biomarker-selected from first-in-human dosing
MRTX1719 (navlimetostat, BMS-986504)/MTA-cooperative PRMT5	MTAP-deletion synthetic lethality	MTAP deletion	Phase I, MTAP-deleted solid tumors incl. mesothelioma and NSCLC (NCT05245500; recruiting) [37]	Early objective responses incl. mesothelioma and NSCLC	No dose-limiting hematologic toxicity up to 800 mg daily; SDMA eliminated in paired biopsies from 3 patients (H-score 240–280 before treatment, 0 on treatment)	Promising but preliminary: early responses
MRTX1719 (navlimetostat, BMS-986504)/MTA-cooperative PRMT5	MTAP-deletion synthetic lethality	Homozygous MTAP deletion	MountainTAP-29 randomized phase 2/3, +pembrolizumab + chemotherapy vs. placebo, 1L metastatic NSCLC (NCT07063745; recruiting)	Pending	Pending	Promising but preliminary: first randomized trial in NSCLC recruiting
TNG462; TNG908/PRMT5 (MTAP-deleted tumors)	MTAP-deletion synthetic lethality	MTAP deletion	TNG462 phase I/II incl. pembrolizumab combination (NCT05732831; recruiting); TNG908 phase I/II (NCT05275478; terminated, sponsor business decision, *n* = 110)	No peer-reviewed efficacy report	Not reported	Insufficiently supported to date: no efficacy report; TNG908 discontinued for business reasons

## 11. Future Directions and Unified Knowledge Taxonomy

Advances in NMR spectroscopy, mass spectrometry, stable-isotope tracing, and computational analysis have greatly expanded the capacity to characterize cancer metabolism, but most oncology applications remain investigational [15,16,20]. Results still depend on the instrument, acquisition method, preprocessing pipeline, and reference libraries used, and integrating metabolomic with genomic, imaging, and clinical data remains difficult because these datasets differ in coverage, measurement scale, missing-data structure, and annotation confidence [16,21,90].

To organize these heterogeneous data, the U.S. National Research Council proposed a Knowledge Network of Disease built on an underlying Information Commons that would connect molecular, clinical, environmental, and population-level information [100]. Rather than replacing conventional histopathologic classification, this evolving framework was intended to support a more biologically informed taxonomy of disease that could be continuously refined as new evidence becomes available [100]. In thoracic oncology, such an infrastructure could integrate histology, oncogenic drivers, metabolite abundances, isotope-derived pathway activity, spatial and single-cell measurements, imaging phenotypes, treatment exposures, and longitudinal clinical outcomes. This approach could improve reproducible disease stratification and generate mechanistic hypotheses. However, data integration alone cannot distinguish causal regulation from correlation; causal conclusions still require experimental perturbation, appropriately designed isotope-tracing studies, temporal measurements, and mechanistic or computational validation [27].

Natural compounds represent a further, exploratory route to probing tumor metabolic dependence. They are widely studied in lung cancer, most often as sensitizers combined with chemotherapy, radiotherapy, targeted therapy, or immunotherapy, but this literature remains predominantly pharmacological and preclinical, and its translation depends on standardized composition, batch-to-batch consistency, pharmacokinetic and bioavailability evaluation, and validation of target engagement in vivo [101]. Within the framework of this review, such compounds are best regarded as candidate tools for interrogating metabolic dependence rather than as therapies. Their metabolic effects can be evaluated with the approaches described in Section 2: steady-state metabolomics to identify perturbed pathways, and stable-isotope tracing, for example with [U-^13^C]glucose or [U-^13^C_5_]glutamine, to determine whether a compound alters glucose, amino-acid, or redox-related pathway activity rather than metabolite abundance alone [19,27]. Distinguishing specific metabolic regulation from non-specific cytotoxicity additionally requires validation of the proposed molecular target, metabolic rescue experiments in which supplying the depleted product or bypassing the inhibited step restores growth, and parallel testing in non-malignant cells at matched exposures. Because many natural products are chemically complex and have limited oral bioavailability, these experiments should use chemically standardized preparations at concentrations achievable in patient plasma or tissue. This should be regarded as an expandable research direction rather than evidence of clinical efficacy.

Several limitations constrain the evidence reviewed here. Thoracic metabolomics studies frequently involve small, retrospective, single-center cohorts; heterogeneous specimen types and handling procedures; variable analytical platforms; incomplete metabolite annotation; and limited independent or prospective validation [15,16,18,20,21,90]. Circulating profiles are influenced by diet, medications, comorbidities, microbiome activity, and systemic organ metabolism and therefore cannot automatically be interpreted as tumor-derived. Smoking, obesity and metabolic disease, and renal or hepatic impairment shift these concentrations further, independently of tumor biology; differences between experimental models and human tumors, intratumoral heterogeneity, and the distinct contributions of stromal and immune cells compound this problem (Table 5). Similarly, steady-state metabolite abundances do not establish pathway flux, and quantitative in vivo flux measurements in patients remain comparatively limited [27]. Predictive models developed from high-dimensional datasets with small sample sizes are also vulnerable to overfitting and poor transportability across institutions and populations. Future investigations should therefore prioritize prospective multicenter cohorts, harmonized pre-analytical and analytical procedures, standardized reference and quality-control materials, preregistered computational pipelines, blinded external validation, paired tissue and biofluid sampling, longitudinal measurements, and stable-isotope tracing where feasible. Clinical adoption will additionally require evidence that metabolomic measurements provide reproducible and cost-effective value beyond established diagnostic, prognostic, and predictive methods. Ultimately, progress will depend not on any single analytical platform or algorithm, but on interoperable data systems and prospective evidence connecting metabolic measurements to clinically meaningful outcomes.

## 12. Conclusions

Metabolomics and isotope-based methods have clarified how thoracic tumors use nutrients, but their clinical role remains limited, and the strength of evidence differs sharply across applications. Only one metabolic assay, ^18^F-FDG PET, is in routine thoracic practice, and only one agent directed at a tumor-specific metabolic dependency, pegargiminase, has improved overall survival in a randomized trial in non-epithelioid pleural mesothelioma, a population enriched for ASS1 deficiency. Promising but preliminary findings include MTA-cooperative PRMT5 inhibition in MTAP-deleted tumors, now in randomized testing in NSCLC; the association of STK11/LKB1 and KEAP1 alterations with poorer outcomes of PD-1/PD-L1 blockade, which rests on retrospective cohorts; and circulating biomarker panels validated only in retrospective case–control studies. Histology-specific dependencies in LUSC and SCLC, and metabolic alterations in esophageal, pleural, and thymic tumors, remain preclinical or hypothesis-generating. Moving these findings toward patients will require prospective multicenter cohorts with standardized pre-analytical and analytical procedures and shared data, paired tissue and blood sampling before and during treatment, isotope tracing where feasible, validation in independent populations, and trials that select patients on a demonstrated metabolic dependency.

## Figures and Tables

**Table 1:** Metabolic-analysis modalities referred to in this review, with what each measures and what it does and does not establish. These terms are used consistently throughout; in particular, metabolite abundance alone does not establish pathway activity or metabolic flux.

Approach	What It Measures	What It Can Establish	Key Limitation
Steady-state metabolomics (untargeted or targeted)	Relative or absolute metabolite pool sizes	Pathway-associated differences; candidate biomarkers	Abundance does not establish pathway activity or flux
Stable isotope–resolved metabolomics (SIRM)	Incorporation of labeled atoms into downstream metabolites	Qualitative substrate fate and pathway utilization	Enrichment alone does not yield absolute flux
^13^C metabolic flux analysis (^13^C-MFA)	Isotope-labeling data fitted to a network model	Quantitative intracellular pathway fluxes	Requires tracer design, modeling, and often invasive sampling
Mass spectrometry imaging	Spatially resolved metabolite distribution in tissue	Compartment-level localization of metabolites	Semi-quantitative; narrower coverage; no flux
Metabolic positron emission tomography (PET) imaging (e.g., ^18^F-fluorodeoxyglucose [FDG])	In vivo radiotracer uptake	Whole-body in vivo metabolic uptake	Limited pathway specificity; uptake is not flux
Hyperpolarized ^13^C magnetic resonance (MR)	Real-time substrate-to-product conversion in vivo	Apparent conversion/exchange rates (e.g., pyruvate → lactate)	Reflects label exchange, not net flux; technically demanding; not established in thoracic tumors
Metabolic biomarker	A measured metabolite or panel linked to a clinical endpoint	Potential diagnostic, prognostic, or predictive signal	Requires validation; is an association, not a mechanism

**Table 2:** Representative metabolomic studies in thoracic malignancies, covering biofluid biomarker studies, the largest spatially resolved tissue studies, and the human in vivo isotope-tracing studies, summarizing population, sample size, specimen and analytical platform, principal finding or diagnostic performance, confounder adjustment and analytical quality control, validation status and incremental value over existing clinical information, and the evidence status assigned using the classification in Section 1. AUC, area under the receiver-operating-characteristic curve; BALF, bronchoalveolar lavage fluid; CA 15-3, carbohydrate antigen 15-3; CA 19-9, carbohydrate antigen 19-9; CEA, carcinoembryonic antigen; CT, computed tomography; CYFRA21-1, cytokeratin 19 fragment; HR, hazard ratio; IAC, invasive adenocarcinoma; LN+, lymph-node positive; MS, mass spectrometry; PDX, patient-derived xenograft; QC, quality control; TCGA, The Cancer Genome Atlas; UICC, Union for International Cancer Control; ↑, increased.

Study [Ref]	Setting/Population	*n*	Specimen & Platform	Key Finding/Performance	Confounding & Analytical QC	Validation; Incremental Value	Evidence Status
Mathé 2014 [68]	Lung cancer vs. controls; diagnosis/prognosis	469/536 + 80/78	Urine; LC–MS	Creatine riboside, N-acetylneuraminic acid; also enriched in tumor tissue	Logistic models adjusted for race, sex, interview year, smoking status, pack-years and urine collection time; survival models also for stage, histology and treatment. Randomized injection order; four quality-control sets including 169 pooled samples; internal standards	Independent case–control cohort. Proposed as an adjunct to low-dose CT for indeterminate nodules, but not tested against a validated risk model	Promising but preliminary (retrospective)
Zhang 2025 [67]	NSCLC early detection	680 + 216	Plasma; targeted LC–MS	10-metabolite model; AUC ≈ 0.92 (stage I–II), ≈0.89 (stage I) with smoking	Age alone was not discriminating (AUC 0.57, *p* = 0.61); smoking (pack-years) alone gave AUC 0.82 and was retained as a cofactor. Isotopically labeled internal standards, calibration curves and quality-control standards	Independent validation set (retrospective). Metabolites alone gave AUC 0.91/0.89 (discovery/validation) and 0.94/0.93 with smoking added, exceeding smoking alone (0.82)	Promising but preliminary (retrospective)
Callejón-Leblic 2019 [82]	Lung cancer; multi-biofluid	32/29; BALF 24/30	Serum/urine/BALF; GC–MS	Amino-acid pathways; only 4 alterations shared across matrices	No adjustment reported. Seven pooled quality-control samples per matrix with stability verified by principal component analysis; locally weighted normalization	None. Single-metabolite AUCs 0.53–0.83; no multivariable model and no comparison with CEA or CYFRA21-1	Hypothesis-generating
Nie 2021 [83]	Advanced NSCLC; anti–PD-1 response	43 (+21, +10)	Serum; MS	Hypoxanthine + histidine predict response/PFS/OS	Responders and non-responders did not differ in age, sex, stage, smoking history, or prior treatment; multivariable Cox regression for survival. Pooled quality-control samples clustered on principal component analysis; internal standard	Small sets; different agent (tislelizumab). AUC 0.972 (discovery) and 0.933 (validation set 1), but no comparison with PD-L1 expression or tumor mutational burden	Hypothesis-generating; HRs likely inflated
Nie 2021 [84]	Lung preneoplasia → IAC progression	181 tissue; plasma 10/32/22/28	Tissue & plasma; targeted LC–MS	Stage-associated shifts; bile-acid-high subtype III; subtypes II and III adverse	Cross-sectional comparison across histological stages; no adjustment. Tissue analyzed in 14 randomized batches with paired samples and quality-control samples inserted in each; plasma in two batches	Cross-sectional; separate cohorts. Four-metabolite panel AUC 0.894 (benign vs. adenocarcinoma); no comparison with CT nodule-risk models	Hypothesis-generating
Wang 2023 [76]	LUSC; prognosis and chemotherapy response	330 resected + 40 neoadjuvant	Tissue; high-mass-resolution MS imaging	4 tumor and 4 stroma metabolic subtypes; one tumor subtype independently prognostic; one stroma subtype linked to chemoresistance	Multivariable Cox regression against UICC stage (tumor subtype T3, HR 0.439, *p* = 0.021). Analytical quality control not reported	Independent neoadjuvant-treated cohort (*n* = 40; chemoresistance). Prognostic value independent of UICC stage, but no comparison with established prognostic models	Promising but preliminary (retrospective)
Zhao 2024 [70]	ESCC early detection	560 participants/1153 samples (3 centers)	Tissue/serum/urine; NMR + MS	Serum 5-metabolite AUC 0.984; urine 0.930; center-3 test	Tissue, serum and urine matched within participants; no further adjustment reported. NMR selected for inter-laboratory reproducibility; sample size set by prospective power calculation	Internal multicenter; not prospective. Five-metabolite serum panel AUC 0.984, against CEA, CYFRA21-1, CA 19-9, CA 15-3 and squamous cell carcinoma antigen, which the authors report as insufficiently sensitive and specific	Promising but preliminary (retrospective)
Huang 2024 [85]	ESCC lymph-node metastasis	tissue 21; plasma 130	Tissue & plasma metabolomics	1-methylnicotinamide ↑ in LN+; test AUC 0.839 (0.872 + T stage)	Age, sex, smoking, alcohol intake, tumor history and T stage were modeled explicitly as a clinical comparator. Targeted peak areas normalized to internal standards	Internal split only. In the 51-patient test set, clinical model AUC 0.711, metabolite model 0.839 and integrated model 0.872, a direct estimate of incremental value	Hypothesis-generating
Sun 2019 [65]	ESCC; spatially localized candidate targets	256 patients	Tissue; airflow-assisted desorption electrospray ionization MS imaging	Dysregulated proline, glutamine, uridine, histidine, fatty-acid, and polyamine pathways; PYCR2, GLS, UPP1 nominated	Matched tumor and normal tissue from the same patients; no adjustment. Analytical quality control not reported	No functional validation in patients; immunohistochemistry confirmed expression of the nominated enzymes in situ, but no outcome or performance estimate	Hypothesis-generating
Di Gregorio 2021 [72]	Pleural mesothelioma; pre/post radiotherapy	19	Serum; targeted LC–MS/MS	Arginine/polyamine pathways perturbed with outcome	Paired pre- and post-treatment design; no adjustment. Quality controls at three concentrations, isotopically labeled internal standards and calibration curves	None; internal leave-one-out cross-validation only, with no comparison against established prognostic factors	Hypothesis-generating
Chen 2021 [12]	Mesothelioma PDX models	5 (2 engrafted)	Mouse serum; GC–MS	Kynurenine, uridine, uric acid, inosine ↑ (xenograft host)	Not applicable (model derived from 5 confirmed cases). Analytical quality control not reported	None	Preclinical
Alwahsh 2022 [10]	Thymic epithelial tumors	15 tumors + 4 normal	Tissue; HRMAS ^1^H-NMR	Lactate/glutathione/redox separate aggressive vs. indolent	No adjustment. Internal standard used for NMR referencing; the authors note that no independent frozen-tissue cohort was available, which may permit false-positive enrichment results	None. Transcriptional cross-check against TCGA thymic epithelial tumors (*n* = 115); no clinical performance estimate	Hypothesis-generating
Miller 2024 [71]	Suspected thymoma; surgical	20 (9 thymoma/11 cyst); ^13^C in 11	Tissue; MS + intraoperative ^13^C	Distinguished thymoma; central-carbon labeling differs	No adjustment; prospective single-arm design (NCT02095808) with intraoperative ^13^C-glucose infusion, not powered for diagnostic accuracy	Small; internal. Thymoma versus benign cyst separation only, with no validation cohort and no comparison with imaging	Hypothesis-generating
Hensley 2016 [25]	NSCLC; in vivo glucose metabolism	9	Intraoperative [U-^13^C]glucose; tumor vs. benign lung	Enhanced glycolysis and glucose oxidation common, but marked inter- and intratumoral heterogeneity; perfusion correlated with glucose contribution to the TCA cycle	Each tumor compared with the patient’s own benign lung; bolus and continuous infusion compared directly	Not applicable (mechanistic study, no biomarker claim)	Hypothesis-generating (human in vivo mechanism)
Faubert 2017 [24]	NSCLC; substrate use in vivo	30 infused with [U-^13^C]glucose, plus ^13^C-lactate infusions	Intraoperative [U-^13^C]glucose and [U-^13^C]lactate	Circulating lactate contributes carbon to the TCA cycle, in many tumors exceeding glucose; MCT1 required for lactate uptake.	Paired tumor and lung; mechanism confirmed causally in xenografts by MCT1 inhibition.	Not applicable; mechanism validated in models rather than against a clinical endpoint	Hypothesis-generating (human in vivo mechanism)
Cai 2025 [26]	NSCLC; tracing linked to outcome	>90 infused over 11 years (66 treatment-naive NSCLC with [U-^13^C]glucose; 13 with ^13^C-lactate)	Intraoperative ^13^C-labeled nutrients	High ^13^C enrichment of TCA-cycle intermediates associated with metastasis and early death; blocking the electron transport chain suppressed metastasis in mice	Single-program cohort accrued over 11 years; no adjustment reported	No independent cohort; causal validation in patient-derived models, not in patients	Promising but preliminary (prognostic association)

**Table 3:** Pre-analytical and analytical variables affecting metabolomic measurements, with recommended control or reporting and the governing standards. QC, quality control.

Variable	Recommended Control/Reporting	Standard/Reference
Fasting and collection time	Prespecify and standardize across participants	ISO 23118:2021 [88]
Blood tube and anticoagulant	Prespecify; keep consistent within a study	[18,88]
Processing delay and temperature	Minimize and prespecify maximum delay before centrifugation	[18,86,87]
Hemolysis	Assess and flag or exclude affected samples	[86]
Freeze–thaw cycles	Limit and record; aliquot at first processing	[18]
Storage	Validated low-temperature storage (commonly −80 °C)	[18,88]
Batch effects and injection order	Randomize or balance; distribute pooled QC samples throughout; report the design	mQACC [80]
Internal standards and system suitability	Report internal standards; system-suitability checks, and predefined acceptance criteria	[80]
Metabolite identification confidence	Report annotation confidence level (five-level high-resolution MS scheme [21,75]; the original Metabolomics Standards Initiative standards [89] define four levels)	[21,75,89]
Inter-laboratory reproducibility	Harmonize workflows; use reference materials	[80,90]
External validation	Independent, ideally prospective, cohort	[15,20]

**Table 5:** Translational barriers to the clinical adoption of metabolomics in thoracic oncology, with representative mitigations. FDG-PET, ^18^F-fluorodeoxyglucose positron emission tomography; SIRM, stable isotope–resolved metabolomics.

Domain	Specific Barrier	Mitigation
Biological confounding	Diet and fasting, gut microbiome, medications, comorbidity, smoking, obesity, metabolic disease, renal/hepatic function, systemic organ metabolism	Capture and adjust for covariates; paired tissue sampling.
Tumor biology	Intratumoral heterogeneity; stromal and immune contributions; differences between models and human tumors	Spatial and single-cell profiling; paired designs; in-patient isotope tracing
Abundance vs. flux	Metabolite pool size does not establish pathway activity	SIRM/^13^C-MFA; in vivo tracing (Section 2 and Section 6.4)
Pre-analytical/analytical	Collection, processing, storage, hemolysis, batch effects, identification confidence	Standardization per Table 3 (ISO 23118, mQACC, Metabolomics Standards Initiative levels)
Statistical/computational	High dimensionality, multiple testing, overfitting, data leakage, batch correction	Section 6.5; prespecified pipelines; TRIPOD+AI reporting
Validation	Small, single-center, retrospective cohorts; limited external or prospective validation	Multicenter prospective cohorts; preregistration; blinded external validation
Clinical value	Added value beyond FDG-PET and established markers unproven	Head-to-head comparison with standard assays; cost-effectiveness analysis

## Data Availability

No new data were created or analyzed in this study. Data sharing is not applicable to this article.

## Abbreviations

The following abbreviations are used in this manuscript:

^1^H-NMR	proton nuclear magnetic resonance
2-DG	2-deoxy-D-glucose
^13^C-MFA	^13^C metabolic flux analysis
1L	first line
AAH	atypical adenomatous hyperplasia
ADI-PEG20	pegylated arginine deiminase (pegargiminase)
ADORA2A	adenosine A2A receptor
ADORA2B	adenosine A2B receptor
AE	adverse event
AIS	adenocarcinoma in situ
AKT	AKT serine/threonine kinase (protein kinase B)
AMPK	AMP-activated protein kinase
ASCL1	achaete-scute family bHLH transcription factor 1
ASS1	argininosuccinate synthetase 1
ATP	adenosine triphosphate
AUC	area under the receiver-operating-characteristic curve
BALF	bronchoalveolar lavage fluid
CA 15-3	carbohydrate antigen 15-3
CA 19-9	carbohydrate antigen 19-9
CAA	cancer-associated adipocyte
CAF	cancer-associated fibroblast
CD4	cluster of differentiation 4
CD8	cluster of differentiation 8
CD36	cluster of differentiation 36 (fatty acid translocase)
CD39	ectonucleoside triphosphate diphosphohydrolase-1 (ENTPD1)
CD44	cluster of differentiation 44
CD73	ecto-5′-nucleotidase (NT5E)
CDKN2A	cyclin-dependent kinase inhibitor 2A
CEA	carcinoembryonic antigen
CI	confidence interval
CT	computed tomography
ctDNA	circulating tumor DNA
CXCL13	C-X-C motif chemokine ligand 13
CYFRA21-1	cytokeratin 19 fragment
Da	dalton
DCR	disease-control rate
DDA	data-dependent acquisition
DESI	desorption electrospray ionization
DIA	data-independent acquisition
EAC	esophageal adenocarcinoma
EGFR	epidermal growth factor receptor
ESCC	esophageal squamous cell carcinoma
FDG	fluorodeoxyglucose
FTICR	Fourier transform ion cyclotron resonance.
GC–MS	gas chromatography–mass spectrometry
GLS	glutaminase
GLUT1	glucose transporter 1 (SLC2A1)
GLUT3	glucose transporter 3 (SLC2A3)
GPR132	G protein-coupled receptor 132
GTP	guanosine triphosphate
H3K18	histone H3 lysine 18
HIF-1α	hypoxia-inducible factor-1α
HK2	hexokinase 2
HK3	hexokinase 3
HMDB	Human Metabolome Database
HR	hazard ratio
HRMAS	high-resolution magic-angle spinning
IAC	invasive adenocarcinoma
IDO	indoleamine 2,3-dioxygenase
IDO1	indoleamine 2,3-dioxygenase 1
IFN-γ	interferon-γ
IHC	immunohistochemistry
IMPDH	inosine monophosphate dehydrogenase
IMPDH1	inosine monophosphate dehydrogenase 1
IMPDH2	inosine monophosphate dehydrogenase 2
ISO	International Organization for Standardization
KEAP1	Kelch-like ECH-associated protein 1
KEGG	Kyoto Encyclopedia of Genes and Genomes
KRAS	Kirsten rat sarcoma viral oncogene homolog
LC–MS	liquid chromatography–mass spectrometry
LC–MS/MS	liquid chromatography–tandem mass spectrometry
LDCT	low-dose computed tomography
LDH	lactate dehydrogenase
LDHA	lactate dehydrogenase A
LDHB	lactate dehydrogenase B
LGALS9	galectin-9
LKB1	liver kinase B1 (STK11)
LN	lymph node
LRRC15	leucine-rich repeat containing 15
LUAD	lung adenocarcinoma
LUSC	lung squamous cell carcinoma
MALDI	matrix-assisted laser desorption/ionization
MAT2A	methionine adenosyltransferase 2A
MCT1	monocarboxylate transporter 1 (SLC16A1)
MCT4	monocarboxylate transporter 4 (SLC16A3)
MDSC	myeloid-derived suppressor cell
MFA	metabolic flux analysis
MIA	minimally invasive adenocarcinoma
MMP11	matrix metallopeptidase 11
mQACC	metabolomics Quality Assurance and Quality Control Consortium
MR	magnetic resonance
MRM	multiple reaction monitoring
MS	mass spectrometry
MS/MS	tandem mass spectrometry
MTA	methylthioadenosine
MTAP	methylthioadenosine phosphorylase
mTOR	mechanistic target of rapamycin
mTORC1	mechanistic target of rapamycin complex 1
MYC	MYC proto-oncogene, bHLH transcription factor
MYCL	MYCL proto-oncogene, bHLH transcription factor (L-Myc)
m/z	mass-to-charge ratio
NEUROD1	neuronal differentiation 1
NFAT1	nuclear factor of activated T cells 1
NK	natural killer
NMR	nuclear magnetic resonance
NRF2	nuclear factor erythroid 2-related factor 2 (NFE2L2)
NSCLC	non-small cell lung cancer
ORR	objective response rate
OS	overall survival
OXPHOS	oxidative phosphorylation
PCA	principal component analysis
PD-1	programmed cell death protein 1
PD-L1	programmed death-ligand 1
PDX	patient-derived xenograft
PET	positron emission tomography
PFK-2	6-phosphofructo-2-kinase
PFKFB3	6-phosphofructo-2-kinase/fructose-2,6-bisphosphatase 3
PFS	progression-free survival
PGK1	phosphoglycerate kinase 1
PI3K	phosphoinositide 3-kinase
PKM	pyruvate kinase M
PKM2	pyruvate kinase M2
PLS-DA	partial least squares–discriminant analysis
PM	pleural mesothelioma
POU2F3	POU class 2 homeobox 3
PPAR	peroxisome proliferator-activated receptor
PR	partial response
PRMT5	protein arginine methyltransferase 5
PYCR2	pyrroline-5-carboxylate reductase 2
QC	quality control
QTc	corrected QT interval
Q-TOF	quadrupole time-of-flight
RAS	rat sarcoma viral oncogene homolog
RB1	RB transcriptional corepressor 1 (retinoblastoma 1)
RCC	renal cell carcinoma
RCT	randomized controlled trial
RNA	ribonucleic acid
RP2D	recommended phase II dose
SANRA	Scale for the Assessment of Narrative Review Articles
SCLC	small cell lung cancer
SD	stable disease
SDMA	symmetric dimethylarginine
SIRM	stable isotope-resolved metabolomics
SLC2A1	solute carrier family 2 member 1 (GLUT1)
SLC16A1	solute carrier family 16 member 1 (MCT1)
SPP1	secreted phosphoprotein 1 (osteopontin) stimulator of interferon genes
STK11	serine/threonine kinase 11 (LKB1)
SUV	standardized uptake value
TCA	tricarboxylic acid
TCGA	The Cancer Genome Atlas
TDO	tryptophan 2,3-dioxygenase
TET	thymic epithelial tumor
TGF-β	transforming growth factor-β
TGR5	Takeda G protein-coupled receptor 5 (GPBAR1)
TME	tumor microenvironment
TNFRSF4	tumor necrosis factor receptor superfamily member 4 (OX40)
TP53	tumor protein p53
Treg	regulatory T cell
TRIPOD+AI	Transparent Reporting of a multivariable prediction model for Individual Prognosis Or Diagnosis, updated for artificial intelligence.
UICC	Union for International Cancer Control
UPP1	uridine phosphorylase 1
VEGFA	vascular endothelial growth factor A
YAP1	Yes-associated protein 1

## Appendix A. PubMed Search Strategy

The searches summarized in Section 1 were run in PubMed/MEDLINE with the strings in Table A1. Each concept string (S1–S4) was combined with the thoracic-malignancy block (T) using AND, and the results were supplemented by screening the reference lists of included articles and key reviews.

**Table A1:** PubMed/MEDLINE search strings used to identify literature for this review. Limits: English language; publication date 1 January 2000 to October 2026 (final update 10 October 2026). MeSH, Medical Subject Headings; [tiab], title/abstract field; *, PubMed truncation symbol.

String	PubMed Query
T (thoracic malignancies)	(“Lung Neoplasms”[MeSH] OR “lung cancer”[tiab] OR “lung adenocarcinoma”[tiab] OR “lung squamous”[tiab] OR “non-small cell”[tiab] OR “small cell lung”[tiab] OR NSCLC[tiab] OR SCLC[tiab] OR “Esophageal Neoplasms”[MeSH] OR “esophageal cancer”[tiab] OR “oesophageal cancer”[tiab] OR “esophageal squamous”[tiab] OR “esophageal adenocarcinoma”[tiab] OR “Mesothelioma”[MeSH] OR mesothelioma[tiab] OR “Thymus Neoplasms”[MeSH] OR thymoma*[tiab] OR “thymic carcinoma”[tiab] OR “thymic epithelial”[tiab])
S1 (metabolomics, isotope tracing, flux, metabolic imaging)	T AND (“Metabolomics”[MeSH] OR metabolom*[tiab] OR metabonom*[tiab] OR “metabolic profiling”[tiab] OR “metabolite profiling”[tiab] OR “isotope tracing”[tiab] OR “stable isotope”[tiab] OR “isotope-resolved”[tiab] OR “13C”[tiab] OR “metabolic flux”[tiab] OR fluxom*[tiab] OR “mass spectrometry imaging”[tiab] OR “spatial metabolomics”[tiab] OR hyperpolarized[tiab] OR “metabolic imaging”[tiab])
S2 (tumor microenvironment and immunometabolism)	T AND (immunometabol*[tiab] OR ((“Tumor Microenvironment”[MeSH] OR “tumor microenvironment”[tiab] OR “tumour microenvironment”[tiab]) AND (lactate[tiab] OR glycolys*[tiab] OR glutamin*[tiab] OR arginine[tiab] OR methionine[tiab] OR hypoxia[tiab] OR “metabolic reprogramming”[tiab])) OR ((“Immune Checkpoint Inhibitors”[MeSH] OR “PD-1”[tiab] OR immunotherapy[tiab]) AND (STK11[tiab] OR LKB1[tiab] OR KEAP1[tiab] OR lactate[tiab] OR metabol*[tiab] OR microbiome[tiab])))
S3 (metabolic biomarkers)	T AND (metabolom*[tiab] OR metabolite*[tiab]) AND (biomarker*[tiab] OR diagnos*[tiab] OR prognos*[tiab] OR predict*[tiab] OR “early detection”[tiab] OR screening[tiab]) AND (serum[tiab] OR plasma[tiab] OR urine[tiab] OR urinary[tiab] OR “bronchoalveolar lavage”[tiab] OR tissue[tiab])
S4 (metabolism-targeted therapy)	T AND (“2-deoxyglucose”[tiab] OR “2-deoxy-D-glucose”[tiab] OR PFKFB3[tiab] OR “PFK-158”[tiab] OR glutaminase[tiab] OR telaglenastat[tiab] OR “CB-839”[tiab] OR “arginine deiminase”[tiab] OR pegargiminase[tiab] OR “ADI-PEG”[tiab] OR ASS1[tiab] OR “argininosuccinate synthetase”[tiab] OR MTAP[tiab] OR PRMT5[tiab] OR MCT1[tiab] OR AZD3965[tiab] OR IMPDH[tiab] OR “metabolic inhibitor*”[tiab])
